# Single-cell profiling uncovers PTPRG-driven stemness in malignant plasma cells and signatures of treatment failure in multiple myeloma

**DOI:** 10.3389/fimmu.2025.1658028

**Published:** 2025-09-18

**Authors:** Jiewen Tan, Jinman Zhong, Yueping He, Yunman Xu, Chang Chen, Dan Xiong

**Affiliations:** Department of Hematology, The Eighth Affiliated Hospital, Southern Medical University (The First People’s Hospital of Shunde, Foshan), Foshan, China

**Keywords:** multiple myeloma, single-cell RNA sequencing, cancer stemness, PTPRG, immunotherapy resistance

## Abstract

**Background:**

Multiple myeloma (MM) is characterized by extensive intratumoral heterogeneity and complex interactions within the bone marrow microenvironment, yet the cellular and molecular drivers of treatment resistance remain poorly defined. Protein tyrosine phosphatase receptor gamma (PTPRG) has emerged as a candidate tumor suppressor in various malignancies by antagonizing proliferative and survival signaling, but its functional and prognostic relevance in MM has not been established.

**Methods:**

We analyzed 103,171 single‐cell transcriptomes from 18 MM samples (10 optimal responders [OR] and 8 suboptimal responders [SOR] to bortezomib–melphalan–prednisone) to investigate cell‐type composition, malignant plasma cell subclusters, and tumor–microenvironment crosstalk. InferCNV was used to distinguish malignant plasma cells, which were further reclustered and correlated with bulk prognostic phenotypes. Differential expression, pathway enrichment, transcription‐factor activity, pseudotime trajectory, and ligand–receptor interaction analyses were performed. Finally, bulk datasets (GSE9782, GSE2658, MMRF-CoMMpass) and *in vitro* knockdown assays in U266 and NCI-H929 cells were used to validate the prognostic and functional role of PTPRG.

**Results:**

Eleven major cell types were annotated, with plasma cells, T/NK cells, and CD14^+^ monocytes predominating; SOR samples exhibited an expanded plasma‐cell fraction and reduced T/NK, CD14^+^ monocyte, pre-B, and HSPC populations. Among 35,944 malignant plasma cells, five subclusters were defined; one subcluster (MalPlasma3) was enriched in SOR samples and harbored 93.1% of cells associated with poor survival. MalPlasma3 and “worse‐survival” cells showed activation of stemness, E2F/MYC targets, and G2M checkpoint pathways, driven by transcription factors E2F8, E2F7, FOXM1, E2F1, and TIMELESS. Pseudotime analysis revealed a bifurcating differentiation toward a resistant phenotype, accompanied by upregulation of cell‐cycle and proliferation modules. In the OR group, enhanced cytotoxic features in NK, effector, and naïve T cells, along with IGF1–IGF1R and IFNG–IFNGR signaling, suggested a supportive microenvironment. In contrast to the known role as a tumor suppressor in solid and hematologic cancers, our integrative analyses identified PTPRG among seven stemness‐related genes upregulated in MalPlasma3 and poor‐survival cells, which was echoed in the observed reduced cell viability and increased apoptosis in MM cell lines following siRNA‐mediated PTPRG knockdown.

**Conclusions:**

This single‐cell multi‐omic dissection implicates a proliferative, stem‐like MalPlasma3 subcluster and identified PTPRG as a key mediator of drug resistance and poor outcome in MM, offering novel prognostic biomarkers and therapeutic targets.

## 1 Introduction

Multiple Myeloma (MM) is a hematological malignancy characterized by the clonal proliferation of aberrant plasma cells within the bone marrow, leading to osteolytic lesions, renal insufficiency, anemia, and hypercalcemia (1). Despite significant advancements in therapeutic strategies over the past decades, including proteasome inhibitors, immunomodulatory drugs, and monoclonal antibodies, MM remains largely incurable, with a substantial proportion of patients ultimately experiencing multiple cycles of relapse as the tumor acquires resistance to each line of treatment (2, 3). This heterogeneity is observed not only at the genomic and transcriptomic levels within malignant plasma cells but also in the composition and functional state of the surrounding tumor microenvironment (TME) (4, 5).

The interplay between malignant plasma cells and various cellular components of the TME, such as immune cells and stromal cells, is increasingly recognized as a critical determinant of disease progression, treatment response, and the emergence of resistance (6, 7). However, a comprehensive understanding of the specific cell populations, their molecular characteristics, and their interactions that distinguish patients with different responses to therapy is still elusive. In recent years, high-throughput single-cell RNA sequencing (scRNA-seq) has emerged as a powerful approach to dissect the cellular composition of MM at high resolution. By profiling gene expression in individual cells, scRNA-seq can unveil rare malignant subpopulations, distinct differentiation states, and cell–cell interaction networks that are indiscernible in bulk analyses (8), thus offering an unprecedented opportunity to dissect this complexity and enabling the identification of rare cell populations and subtle transcriptional shifts that may underpin therapeutic failure (9, 10). Previous single-cell studies have identified minor subclones of malignant plasma cells with stem cell-like properties in relapsed/refractory myeloma – highly proliferative, therapy-resistant cells with elevated “stemness” gene signatures that are thought to drive disease recurrence (11).

Among the molecular regulators that may contribute to drug resistance in MM, the role of protein tyrosine phosphatases has gained attention (12, 13). In particular, Protein Tyrosine Phosphatase Receptor Type G (PTPRG) stands out as a candidate tumor suppressor and signaling modulator in hematologic malignancies (12, 13). Notably, in chronic myeloid leukemia (CML), PTPRG is significantly downregulated in leukemic cells at diagnosis, and *PTPRG* hypermethylation has been identified as an independent mechanism of resistance to tyrosine kinase inhibitor therapy (12). However, the involvement of PTPRG in plasma cell myeloma has not been well explored, and it remains unclear whether PTPRG dysfunction might promote therapy resistance in MM and involved signaling pathways.

In this study, we utilized single-cell RNA sequencing (scRNA-seq) to comprehensively profile the bone marrow microenvironment of MM patients stratified by their response to treatment. Our primary objectives were to: (i) delineate the cellular heterogeneity of malignant plasma cells and identify subclones associated with poor prognosis and treatment resistance; (ii) characterize the functional states and pathway activities within these aggressive plasma cell populations; (iii) investigate alterations in the immune cell landscape, particularly T/NK cell subsets, between OR and SOR groups; and (iv) elucidate the cell-cell communication networks that may contribute to suboptimal treatment outcomes. By integrating these single-cell insights with bulk transcriptomic data and performing *in vitro* functional validation, we aimed to uncover novel molecular mechanisms and potential therapeutic targets associated with drug resistance in MM.

## 2 Materials and methods

### 2.1 Single cell data

We retrieved the single cell RNA sequencing (scRNA-seq) data from Gene Expression Omnibus via the access number GSE189460 (8), which includes pre-treatment bone marrow specimens from 18 patients with multiple myeloma (MM) who underwent bortezomib–melphalan–prednisone therapy. Based on clinical response, 10 of these 18 samples were optimal responders (OR), while the rest 8 samples were suboptimal responders (SOR).

### 2.2 Bulk transcriptomics data

We also retrieved bulk mRNA-seq data from three studies, including two from GEO and one from MMRF database. Specifically, mRNA-seq data, together with complete survival records, were retrieved from a study of 113 responders (R) and 126 non-responders (NR) who were enrolled in bortezomib (PS-341) clinical trial for MM treatment (8). Two sets of transcriptome data are available given that two Affymetrix platforms (GPL96 [HG-U133A] and GPL97 [HG-U133B]) were applied for mRNA sequencing two sets of transcriptome data are available. Meanwhile, the mRNA-seq data from 558 cases who enrolled in Total Therapy 2 (TT2) and Total Therapy 3 (TT3) was retrieved GEO (GSE2658) (14). In addition, we also obtained mRNA-seq profiles for 764 MM patients from CoMMpass (MMRF) database deposited in the Multiple Myeloma Research Foundation (https://research.themmrf.org).

### 2.3 Single-cell quality control and annotation

Single-cell preprocessing was performed in Seurat v4.1.1. Cells exceeding 10% mitochondrial gene content or 5% hemoglobin gene expression, or expressing fewer than 200 or more than 5,000 genes, were filtered out in line with previous studies (15–17). Doublets were detected and removed using the DoubletFinder package. Batch effects were corrected via the RunHarmony function in the harmony package (18). Prior to differential, enrichment and statistical analyses, data normalization (i.e., log-transformation), clustering, and dimensionality reduction analyses were performed to all scRNA-seq data using Seurat. We use FindVariableFeatures to select genes (n=2000) and conducted principal component analysis (PCA, via RunPCA), in which the top 20 PCs were retained for the following analysis. Cell clustering (resolution: 0.6) was conducted using FindClusters, with the identified clusters being annotated according to marker genes from CellMark2.0 and well-characterized lineage markers.

### 2.4 Functional enrichment

Differentially expressed genes (DEGs) for each cluster were identified using Seurat’s FindMarkers function. In our study, we focused on upregulated genes in downstream analyses. The potential biological pathways of these genes were identified via Gene Ontology (GO) and Kyoto Encyclopedia of Genes and Genomes (KEGG) enrichment analyses using clusterProfiler v4.8.2 (19).

### 2.5 Identification of malignancy status of plasma cell

We applied Infercnvpy with default parameters to distinguish malignant from non-malignant plasma cells. Normal cells from the tumor microenvironment (TME), specifically T/NK cells, were used as the reference population.

### 2.6 Prognosis-associated subcluster detection

We utilized the “Scissor” (v 2.0.0) (20) algorithm to link the survival status of 764 bulk transcriptomic samples with complete transcriptome data (expression data using log2(fpkm+1)) and complete survival status in the MMRF_COMMPASS dataset to single-cell data of multiple myeloma. Patients with OS status of death were classified as worse status, while those with OS status of alive were classified as good status. The Scissor function was run on epithelial cells with the following parameter settings: alpha=0.05, family = “cox”. Scissor+ cells were associated with worse status, while Scissor- cells were associated with good status.

### 2.7 Transcription factor regulatory network analysis

SCENIC analysis was performed using the pySCENIC v0.12.1 pipeline (21) to infer regulon activity scores (RAS) in malignant plasma cells. GRNBoost2 was used to infer co-expression modules of transcription factors (TFs) and candidate targets. RcisTarget identified enriched DNA motifs within these modules, defining each TF and its direct targets as a regulon. Regulon activity per cell was quantified using AUCell.

### 2.8 Pseudotime trajectory inference

Monocle v2.28.0 (22) was used to reconstruct differentiation trajectories among malignant plasma cells. Following dimensionality reduction and cell ordering, cells were mapped onto branched trajectories. Branch Expression Analysis Modeling (BEAM) was then used to identify genes exhibiting branch-dependent expression dynamics, shedding light on fate decision mechanisms.

### 2.9 Cell–cell communication analysis

The CellChat v1.6.1 algorithm (23) was applied to a merged Seurat object containing malignant plasma cells and other TME populations. After constructing the CellChat object with a curated ligand–receptor database, we used computeCommunProb and computeCommunProbPathway to infer the interaction probabilities at both individual receptor–ligand and signaling pathway levels.

### 2.10 Functional scoring with AUCell

We scored malignant plasma cells for “cancer stemness” and T cells for “cytotoxicity” using AUCell. Gene sets were sourced from the Molecular Signatures Database (MsigDB v2023.1; https://www.gsea-msigdb.org/gsea/index.jsp), with the T-cell cytotoxicity signature detailed in **Supplementary Table S1**.

### 2.11 Survival analysis

We dichotomized samples into PTPRG-high and PTPRG-low groups based on the determined cut-offs (via surv_cutpoint in survminer package). The survival rate of these two groups and comparisons were visualized using Kaplan–Meier curves.

### 2.12 Cell culture and siRNA transfection

The multiple myeloma cell lines U266 and NCI-H929 were cultured in RPMI-1640 medium supplemented with 10% fetal bovine serum (Gibco), 100 U/mL penicillin G, and 100 μg/mL streptomycin at 37°C in a humidified incubator containing 5% CO_2_. Cells in the logarithmic growth phase (~4 × 10^5^) were transfected with 5 nM gene-specific siRNA or negative control siRNA (si-NC; GenePharma, Shanghai) using Lipofectamine 3000 (Invitrogen), following the manufacturer’s protocol. Knockdown efficiency was confirmed by quantitative reverse transcription PCR (qRT-PCR); Primer sequences are listed in **Supplementary Table S2**.

### 2.13 RNA extraction and qRT-PCR

Total RNA was extracted from MM cells using TRIzol reagent (Thermo Fisher Scientific) and reverse-transcribed using PrimeScript™ RT (Takara). Quantitative reverse transcription PCR (qRT-PCR) was performed using HiScript II Q RT SuperMix (TRANS, AU341) to assess the expression of target genes, with β-actin used as the internal control. Each experiment included three biological replicates, each with technical triplicates. Primer sequences used in this study are listed in **Supplementary Table S3**.

### 2.14 CCK-8 proliferation assay

We used the CCK8 assay to detect the viability of cells in accordance with the manufacturer’s protocol. U266 and NCI-H929 cells transfected with siNC, siPTPRG-1, and siPTPRG-2 were seeded into 96-well plates at a density of 5,000 cells per well. At 0, 24, 48, 72, and 96 hours, 10 μL of CCK-8 solution (Biosharp, Shanghai, China) was added to each well, followed by incubation for 1 hour at 37°C. Absorbance was then measured at 450 nm using a microplate reader (BD Biosciences, USA). The data were analyzed and visualized using GraphPad Prism software.

### 2.15 Apoptosis assay

Apoptosis was assessed using an Annexin V-FITC/PI apoptosis detection kit (BD Biosciences, Franklin Lakes, NJ, USA). U266 and NCI-H929 cells were harvested 24 hours after siRNA treatment and resuspended in 1× binding buffer. A total of 100 μL of the cell suspension was incubated with 5 μL Annexin V-FITC and 2.5 μL propidium iodide (PI) for 30 minutes at 37°C in the dark. Samples were then analyzed using a BD FACSCanto II flow cytometer (BD Biosciences). Cells positive for Annexin V but negative for PI were considered early apoptotic, while double-positive cells were considered late apoptotic or necrotic. Flow cytometry data were analyzed using FlowJo software (BD Biosciences).

### 2.16 Western blotting

In order to analyze the effect of PTRPG on downstream pathway proteins, cells and tissues were lysed with RIPA lysis buffer containing 1% PMSF. The lysates were then centrifuged, and the supernatant was collected. The quantified protein supernatant was supplemented with 4× protein loading buffer proportionally, boiled for 10 min to denature the protein, and stored at −80°C. Proteins were then separated by 10% SDS-PAGE and electrophoretically transferred onto polyvinylidene fluoride membranes where they were blocked with 5% skim milk and incubated with β-actin (Proteintech, 20536-1-AP, 1:10,000), anti-PTRPG (Abclonal, A14253, 1:1000), caspase-3 (CST, 24232, 1:800), and cleaved caspase-3 (Proteintech, 68773-1, 1:3,000). overnight at 4°C. Next, the membranes were incubated with horseradish peroxidase-conjugated anti-rabbit IgG. Antigen–antibody complexes were then detected with enhanced chemiluminescence reagent. The resulting Images were processed and analyzed using ImageJ software.

### 2.17 Statistical analysis

We performed a minimum of three independent biological replicates for all experiments. Continuous variables were compared using the Mann–Whitney U test (two groups) or Kruskal–Wallis test (more than two groups); categorical variables were assessed by χ² test. All analyses were conducted in R v4.0.5. Two-tailed p < 0.05 was considered statistically significant otherwise stated.

## 3 Results

### 3.1 Cell-type classification and annotation

**Figure 1:**
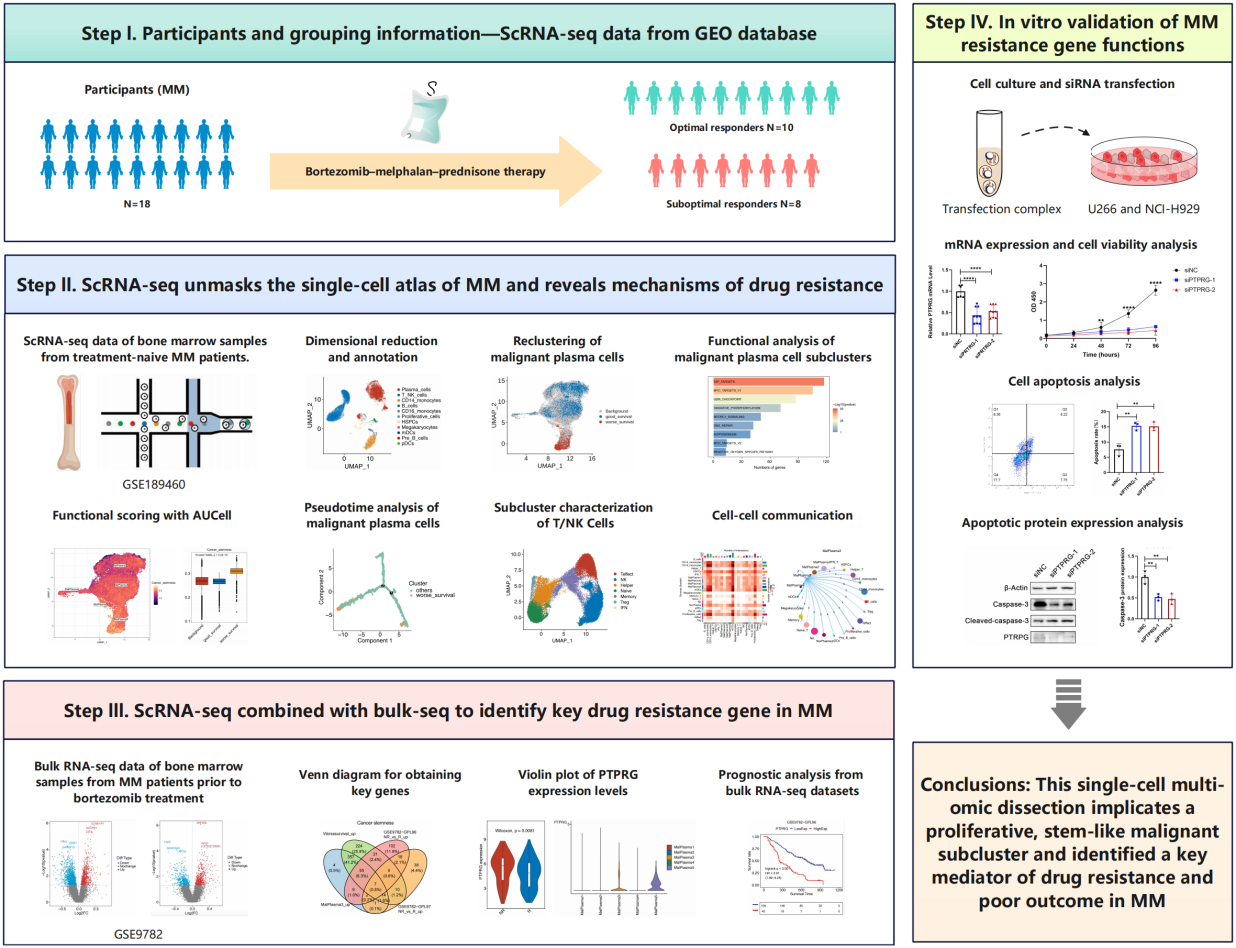
Overview of the study design.

**Figure 2:**
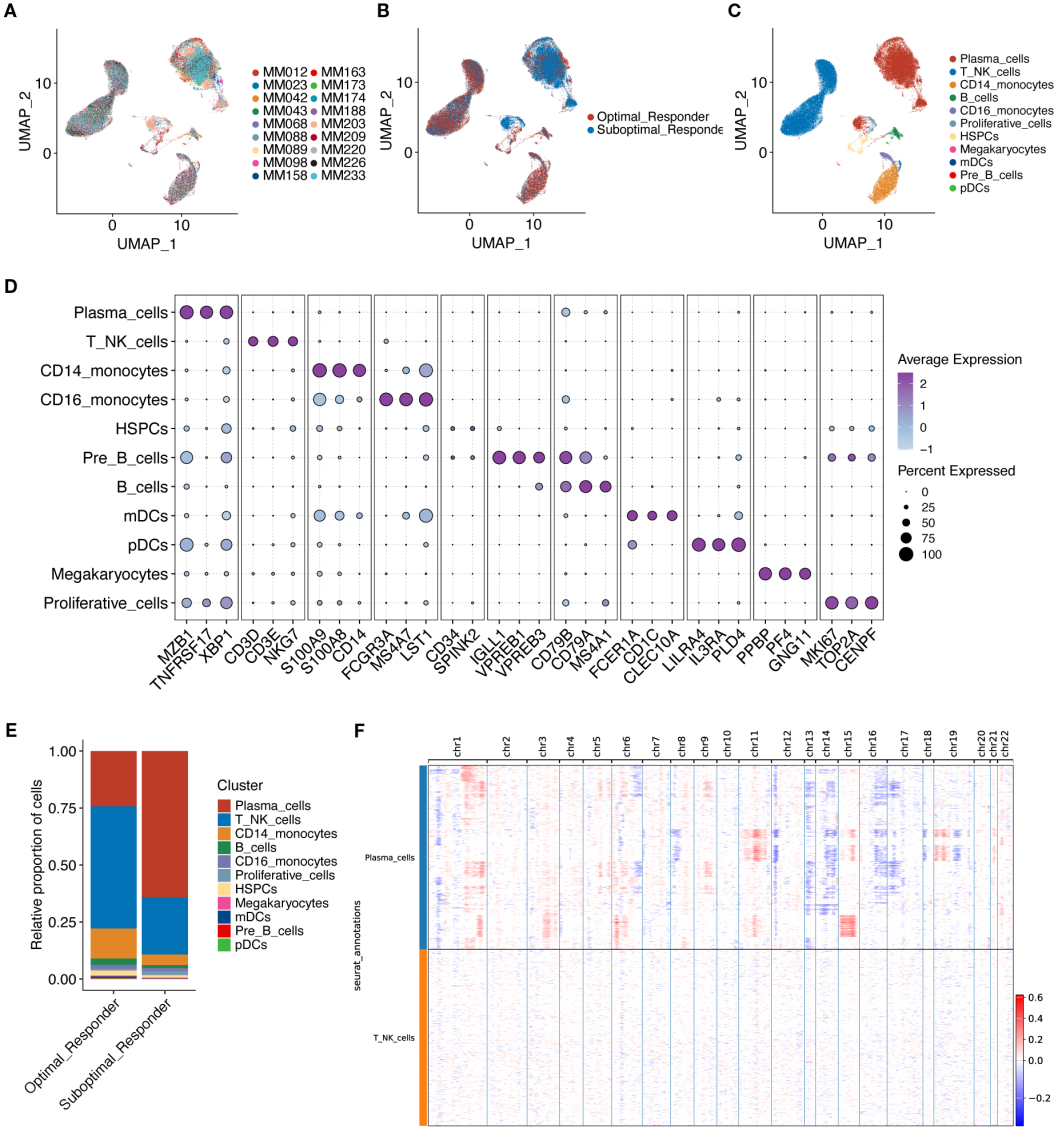
UMAP visualization technology was used for unsupervised clustering analysis of cells, where each dot represents a single cell. **(A)** Cells colored by individual sample. **(B)** Cells colored by treatment response group. **(C)** Cells colored by annotated cell type. **(D)** Shows characteristic marker genes for each cell type. **(E)** Shows the proportion of cells in the two groups. **(F)** Shows large-scale copy number variations (CNVs) in plasma cells via a hierarchical heatmap to identify malignant plasma cells.

A total of 18 samples with single-cell data were obtained from the GSE189460 dataset (**Figure 1**, **Figure 2A**), including 10 OR tissue samples and 8 SOR tissue samples (**Figure 2B**, **Supplementary Table S4**). After data quality control, a total of 103,171 single cells were retained. Dimensionality reduction and clustering analysis identified 27 cell clusters (**Supplementary Figure S1A**). Based on the expression of cell marker genes, 11 cell types were annotated as plasma cells, T/NK cells, CD14+ monocytes, B cells, CD16+ monocytes, Proliferative cells, Hematopoietic Stem and Progenitor Cells (HSPCs), Megakaryocytes, myeloid Dendritic Cells (mDCs), plasmacytoid Dendritic Cells (pDCs), and Pre-B cells (**Figure 2C, D**). The three most abundant cell types were Plasma cells, T/NK cells, and CD14+ monocytes (**Figure 2C**, **Supplementary Figure S1B**). The calculated proportion of each cell type was consistent across examined samples indicating a good data integration (**Supplementary Figure S1C**). Analysis of the changes in the proportion of various cell types between the two groups revealed that the proportion of plasma cells in the SOR group was significantly higher than in the OR group, while the proportions of T/NK cells, CD14+ monocytes, pre-B cells, and HSPCs were significantly lower in the SOR group (**Figure 2E**, **Supplementary Figure S1D**). Furthermore, the copy number variation (CNV) score for each plasma cell was calculated using inferCNV (**Supplementary Figure S1E**), leading to the identification of 35,944 malignant plasma cells (**Figure 2F**, **Supplementary Figure S1F**).

### 3.2 Reclustering of malignant plasma cells

**Figure 3:**
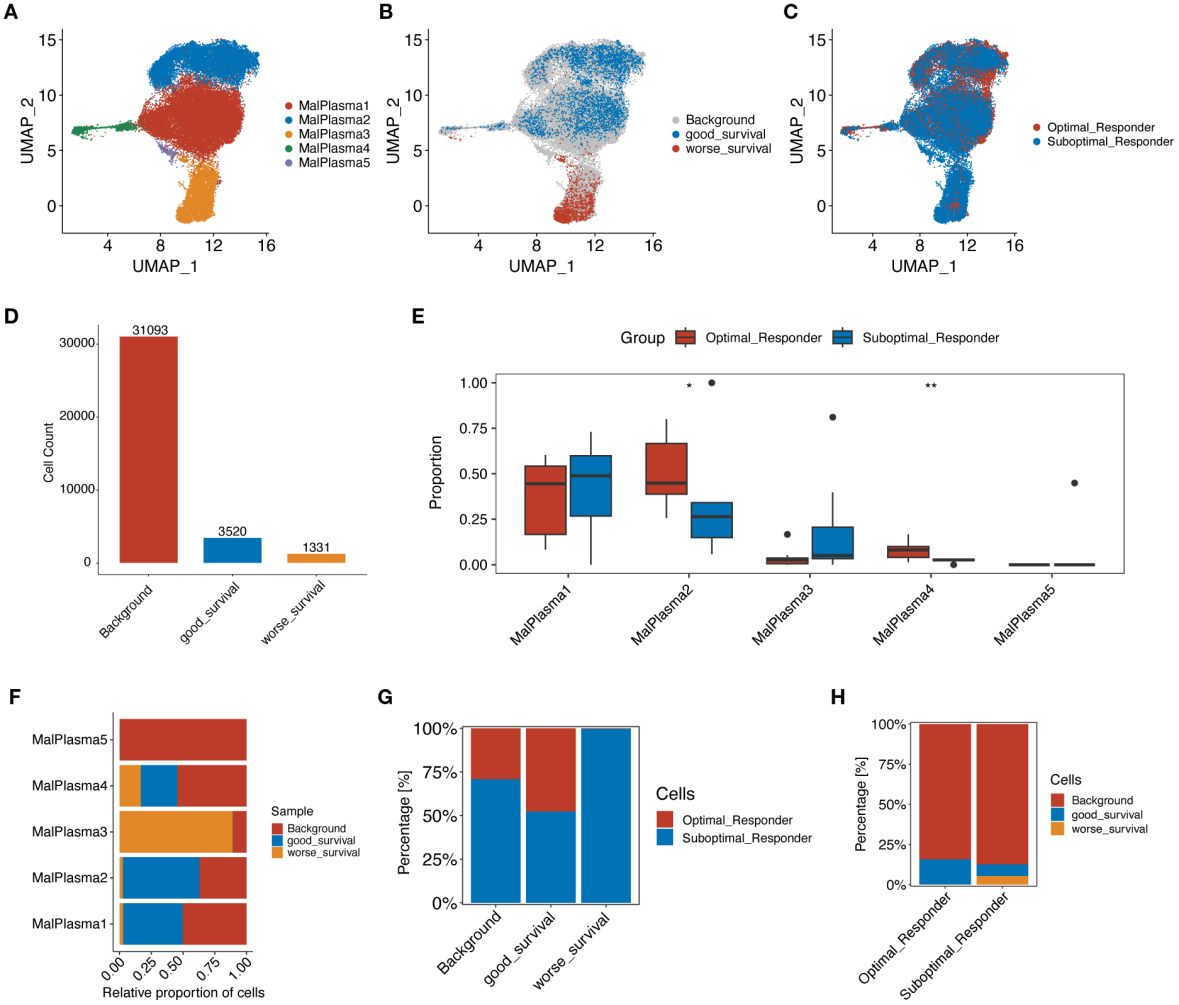
Reclustering analysis of malignant plasma cells. **(A)** UMAP plot showing the distribution of malignant plasma cells, colored by subcluster. **(B)** UMAP plot showing the distribution of malignant plasma cells selected by the Scissor algorithm, classified by prognostic risk and protection. Red and blue dots represent cells associated with poorer and better prognosis phenotypes, respectively. **(C)** UMAP plot showing the distribution of malignant plasma cells, colored by group. **(D)** Number of poor prognosis, good prognosis, and background (prognosis-unrelated) malignant plasma cells. **(E)** Boxplot showing the proportion of origins from the two groups within each malignant plasma cell subcluster. **(F)** Stacked bar plot showing the proportion of poor prognosis, good prognosis, and background malignant plasma cells within each malignant plasma cell subcluster. **(G)** Stacked bar plot showing the proportion of malignant plasma cells from OR and SOR groups within the poor prognosis, good prognosis, and background malignant plasma cell categories. **(H)** Stacked bar plot showing the proportion of poor prognosis, good prognosis, and background malignant plasma cells within the OR and SOR groups.

We then focused on malignant plasma cells to explore the potential heterogeneity. By conducting clustering analyses, the identified malignant plasma cells were reclassified into 5 cell subclusters (**Figure 3A**). By correlating with prognostic phenotypes from bulk transcriptome data, we identified a subcluster containing 1,331 malignant plasma cells associated with poor prognosis (i.e., worse survival) while a subcluster containing 3,520 malignant plasma cells shows good prognosis (i.e., good survival) (**Figure 3B, D**). Cell proportion analysis revealed that in malignant plasma cell subcluster 3 (MalPlasma3), the proportion of cells from the SOR group was higher than that from the OR group (**Figure 3C, E**). Furthermore, 93.1% of the worse survival malignant plasma cells originated from the MalPlasma3 subcluster (**Figure 3F**), indicating that the MalPlasma3 subcluster might be a core driver of poor prognosis, being enriched in the SOR group and highly correlated with treatment resistance. Additionally, worse survival malignant plasma cells were exclusively present in the SOR group (**Figure 3G, H**), suggesting these cells might be the direct cause of treatment failure.

### 3.3 Functional analysis of malignant plasma cell subclusters

**Figure 4:**
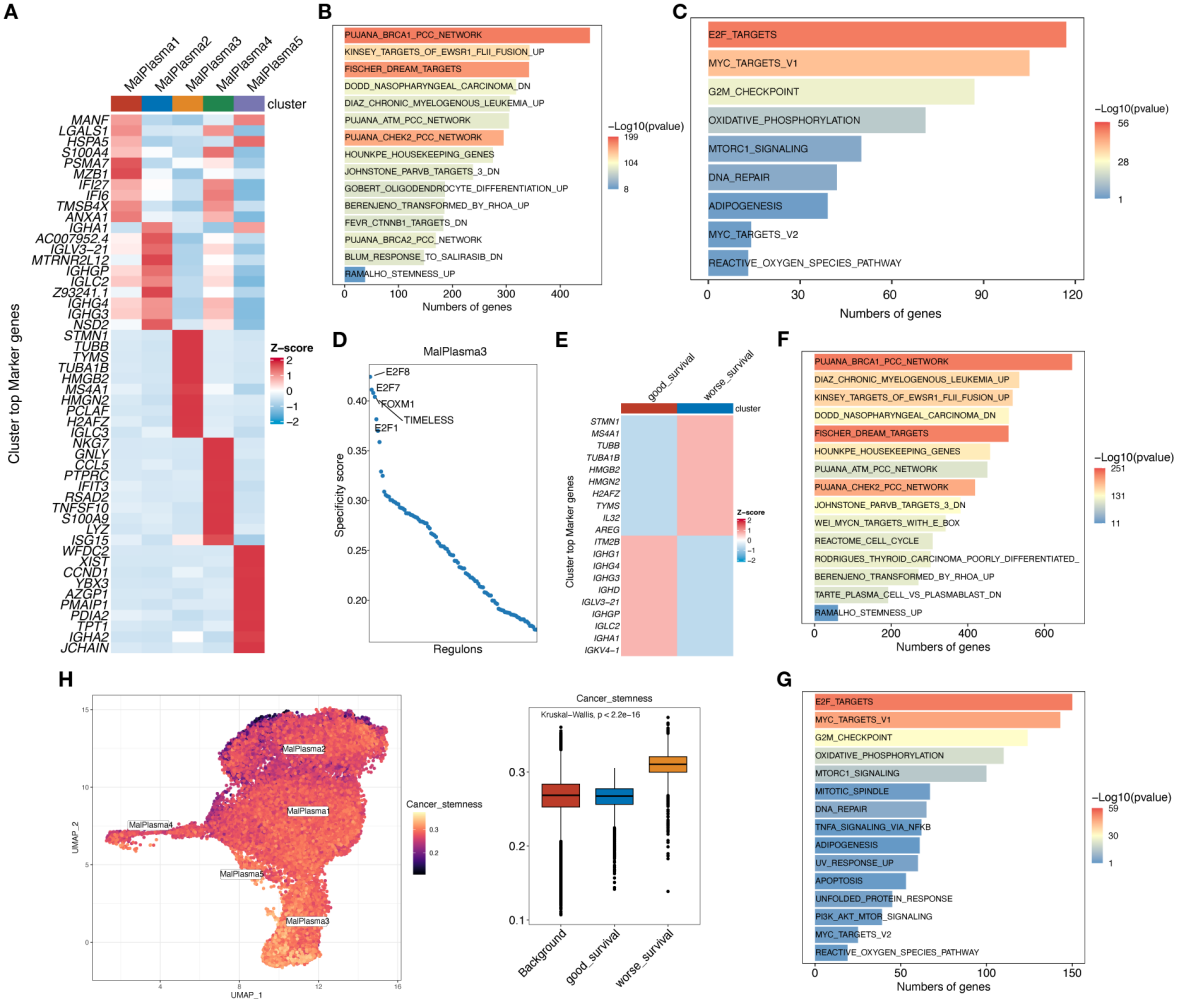
Functional analysis of malignant plasma cell subclusters. **(A)** Heatmap showing the expression levels of the top 10 marker genes for the 5 malignant plasma cell subclusters. **(B)** Bar chart showing the KEGG enrichment analysis results for differentially upregulated genes in malignant cell subtype 3. **(C)** Bar chart showing the HALLMARK enrichment analysis results for differentially expressed genes in malignant cell subtype 3. **(D)** Scatter plot showing the RSSs (regulon specificity score) in poor prognosis malignant cells. The top 5 regulons are highlighted. **(E)** Heatmap showing the expression levels of the top 10 marker genes in worse survival and good survival malignant plasma cells. **(F)** Bar chart showing the KEGG enrichment analysis results for differentially upregulated genes in worse survival malignant plasma cells. **(G)** Bar chart showing the HALLMARK enrichment analysis results for differentially expressed genes in worse survival malignant plasma cells. **(H)** AUCell calculation of the Cancer stemness pathway signature in worse survival, good survival, and background malignant plasma cells. UMAP plot shows the Cancer stemness signature score in each malignant plasma cell; yellower color indicates higher relevant gene expression. Box plot shows the difference in Cancer stemness signature scores among the 3 cell subclusters.

We then conducted differential gene expression analysis to identify genes differing across malignant plasma cell subclusters (**Figure 4A**) and between worse survival and good survival malignant plasma cells (**Figure 4E**) (padj<0.05, |log2FC|>0.25) and conducted functional enrichment analysis to reveal potential pathways these differential genes involved (**Figure 4B, C, F, G**). KEGG results indicated that the Stemness up pathway was significantly activated in the MalPlasma3 subcluster and worse survival malignant plasma cells. HALLMARK results showed significant activation of E2F targets, MYC targets V1/V2, and G2M checkpoint pathways in the MalPlasma3 subcluster and worse survival malignant plasma cells. These pathways are closely related to cell stemness, cell cycle, and cell proliferation and differentiation. These findings collectively suggest that the MalPlasma3 subcluster and worse survival malignant plasma cells play important roles in promoting cell proliferation, differentiation, and invasion, which directly leads to patient drug resistance and poor prognosis. Furthermore, transcription factor analysis (**Figure 4D**) showed that in the malignant cell MalPlasma3 subcluster, the top 5 transcription factors with the highest activity were E2F8, E2F7, FOXM1, E2F1, and TIMELESS, a group of regulators known to be related to cell proliferation, invasion, and cell cycle. These results suggested that worse survival malignant plasma cells exhibit significant characteristics in terms of cell stemness and cycle regulation, leading to poor prognosis. Therefore, AUCELL analysis was further used to assess the activity of the Cancer stemness pathway signature in worse survival, good survival, and background malignant plasma cells. The results showed that the Cancer stemness signature activity was significantly higher in worse survival malignant plasma cells than in good survival and background malignant plasma cells (**Figure 4H**), whereas the differences between good survival and background malignant plasma cells were nonsignificant (**Figure 4H**).

### 3.4 Pseudotime analysis of malignant plasma cells

**Figure 5:**
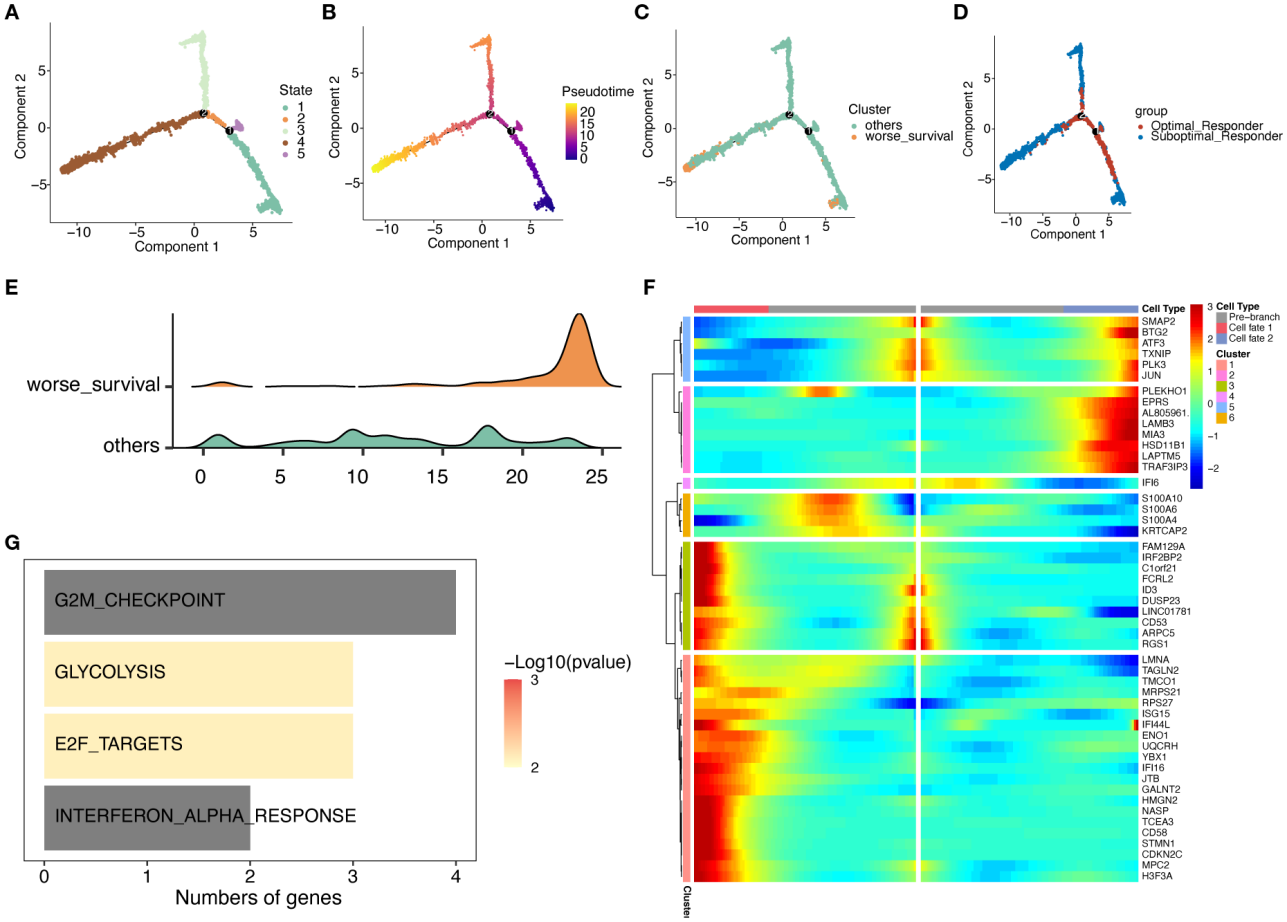
Trajectory analysis of malignant plasma cell subtypes. **(A)** Cell state trajectory (color represents different differentiation states). **(B)** Color represents pseudotime order. **(C)** Cell color represents worse survival and other cells. **(D)** Groups marked by color. **(E)** Cell differentiation ridge plot. **(F)** Heatmap showing dynamic changes in gene expression along pseudotime. **(G)** KEGG enrichment results for Cluster 1 genes.

We then investigated the dynamic evolution process of malignant plasma cells through psuedotime analysis. As shown in Fig4, malignant plasma cells exhibit 5 different states: cells in state 1 were considered potential starting points, followed by a bifurcation at branch point 1, where cells in state 2 developed towards the left of the trajectory, and cells in state 5 developed towards the right (**Figure 5A, B**). Furthermore, we found that more SOR group malignant plasma cells and worse survival malignant plasma cells were located at the terminal end of the differentiation time after branch point 1 as visually documented in cell trajectory plot (**Figure 5C, D**) and ridge plot (**Figure 5E**). Using the branched expression analysis modeling (BEAM), we identified 50 branch-dependent genes that play key roles in regulating cell differentiation from pre-branch to post-branch (Cell fate 1, Cell fate 2). Based on expression similarity, these genes were further divided into 6 modules (clusters). As shown in **Figure 5F**, Cluster 1 exhibited an overall upward trend in gene expression levels during differentiation from pre-branch towards the left of the trajectory after branch point 1 (Cell fate 1). Furthermore, HALLMARK enrichment analysis showed that Cluster 1 genes were mainly enriched in pathways related to cell cycle, proliferation, and differentiation, such as G2M checkpoint and E2F targets (**Figure 5G**). Consistent with the previous differential gene enrichment results, these results further indicate that malignant plasma cells gradually differentiate into a worse survival phenotype, which is accompanied by dramatically enhanced abilities in proliferation, differentiation, and invasion, thus affecting drug efficacy and leading to patient drug resistance.

### 3.5 Subcluster characterization of T/NK Cells

**Figure 6:**
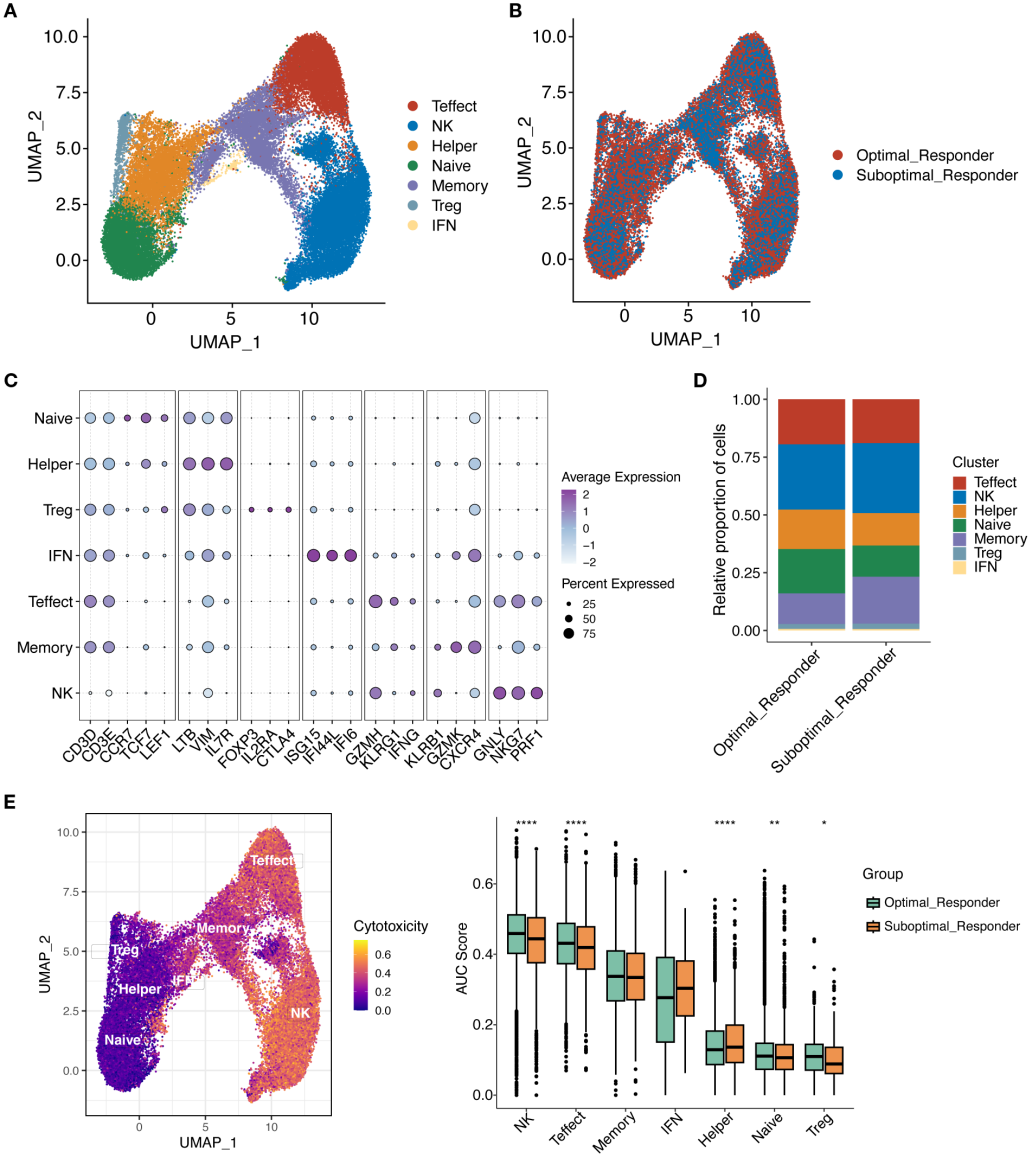
Reclustering of T/NK cells. **(A)** UMAP plot showing the distribution of T/NK cell subclusters. **(B)** UMAP plot showing the distribution of T/NK cell subclusters in OR and SOR groups. **(C)** Dot plot showing the average expression levels of typical marker genes for T/NK cell subclusters. **(D)** Shows the proportion of T/NK cell subclusters in OR and SOR group samples. **(E)** Analysis of T cell cytotoxicity using AUCell scores. UMAP projection showing cytotoxicity scores per T cell (higher scores in yellow indicate increased expression of cytotoxicity-related genes). Box plot demonstrates cytotoxicity signature scores among T cell subtypes across the analyzed sample groups.

To further investigate the potential role of tumor microenvironment (TME) in MM, we further reclustered T/NK cells using unsupervised dimensionality reduction and clustering analysis. We found that T/NK cells were grouped into 12 cell clusters (**Supplementary Figure S2A**). Based on gene expression of cell marker, a total of 7 cell subclusters were identified, including Natural Killer cells (NK), Effector T cells (Effect T), Helper T cells (Th), Naïve T cells, Memory T cells, Regulatory T cells (Treg), and Interferon T cells (IFN T) (**Figure 6A, C**). Notably, all T/NK cell subclusters were shared between the OR and SOR groups, but they exhibited heterogeneous cell proportions (**Supplementary Figure S2B, D-E**). Analysis of cell counts and proportions between these two groups revealed that the top 3 most abundant cell types were NK cells, T-effect cells, and T naïve cells, respectively (**Supplementary Figure S2C**). Furthermore, Naïve T and Th cells showed a significant increase in proportion in the OR group, while Memory T cells showed a significant decrease in proportion in the OR group (**Figure 6B, D**, **Supplementary Figure S2F**). This finding suggests that remodeling of the immune microenvironment may be a key factor for good treatment response. It has been shown that Naïve T cells are unactivated T cells with high proliferative potential and differentiation capacity, while Th cells can enhance anti-tumor immune responses. In the OR group, the increased proportion of Naïve T and Th cells may reflect stronger anti-tumor immune potential and indicate that patients are more sensitive to the bortezomib-melphalan-prednisone regimen. Given that memory T cells have long-term survival and rapid response capabilities to antigens, the significant decrease in the proportion of these cells in the OR group may reflect their effectively activation and differentiation into effector T cells while reduced proportions of Memory T cells during treatment. Further scoring analysis of the cytotoxic features of T/NK cell subclusters (**Figure 6E**) showed that compared to the SOR group, the cytotoxicity feature scores of NK cells, Effect T cells, and Naïve T cells were all significantly elevated in the OR groups, indicating that these cells have stronger cytotoxicity and immune killing effects in the OR group, enabling them to effectively attacking and clearing tumor cells, and exhibiting stronger sensitivity to drugs.

### 3.6 Cell-cell communication

**Figure 7:**
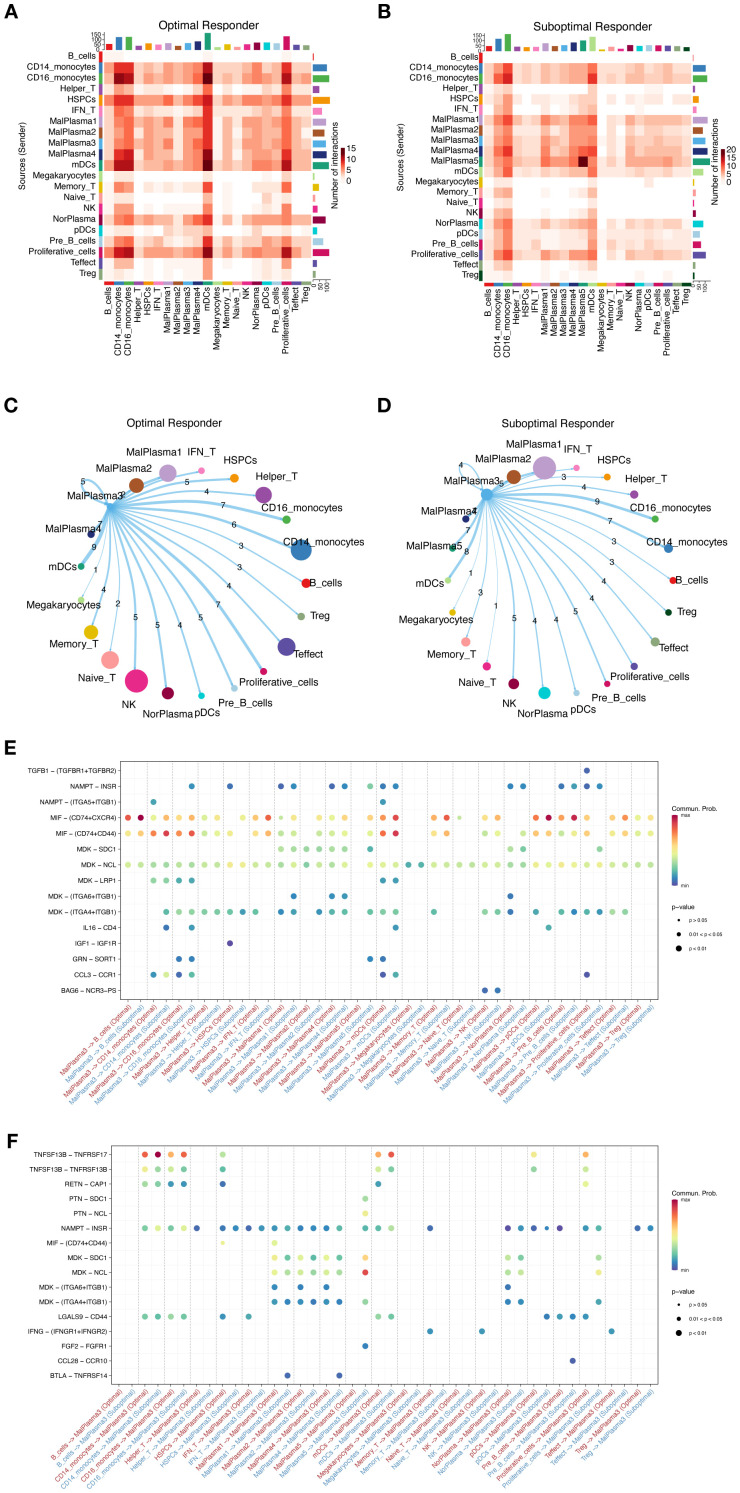
Cell interactions in the MM microenvironment. **(A, B)** Heatmaps showing the overall interaction strength between specific cell subtypes in the OR group **(A)** and SOR group **(B)**. **(C, D)** Chord diagrams showing the interaction network of MalPlasma3 subcluster cells with other cells in the OR group **(C)** and SOR group **(D)**. **(E)** Bubble plot showing the differences in specific ligand-receptor interactions between malignant plasma cells as signal-sending cells and other cells in the OR and SOR groups. **(F)** Bubble plot showing the differences in specific ligand-receptor interactions between malignant plasma cells as signal-receiving cells and other cells in the OR and SOR groups.

To better interpret the communication between cellular components in TME, we constructed cell interaction networks of potential receptor-ligand pairs for the OR and SOR groups, respectively (**Figure 7A, B**). We observed that compared to the OR group, the communication between different cellular components in SOR group samples varied considerably. Given the observed significant activation of pathways related to cell stemness, cell cycle, and differentiation in the MalPlasma3 subcluster and worse survival malignant plasma cells, we thus focused on the interaction of cells in this subcluster with HSPCs. We found that MalPlasma3 subcluster, acting as signal-sending cells, presented significant larger number of interactions with HSPCs in OR groups as compared to the interactions in SOR group (**Figure 7C, D**). Further comparative analysis of ligand-receptor pairs between the two groups revealed that in the OR group, the MalPlasma3 subcluster specifically regulated HSPCs via IGF1-IGF1R, a signaling pathway regulating cell growth, proliferation, and apoptosis by activating signaling pathways such as PI3K/Akt and MAPK (**Figure 7E**). These results were echoed with previously observed increased proportion of HSPCs in the OR group, reflecting a relatively healthier bone marrow microenvironment, but stronger hematopoietic/immune reconstruction ability, and potential anti-tumor immune regulatory effects in this group. In addition, we found that in the OR group, Memory T, NK, and Effect T cells specifically regulated the MalPlasma3 subcluster via IFNG-(IFNGR1+IFNGR2) (**Figure 7F**). Furthermore, in the OR group, Pre-B cells were found to specifically regulate the MalPlasma3 subcluster via CCL28-CCR10.

### 3.7 PTPRG affects prognosis and drug resistance

**Figure 8:**
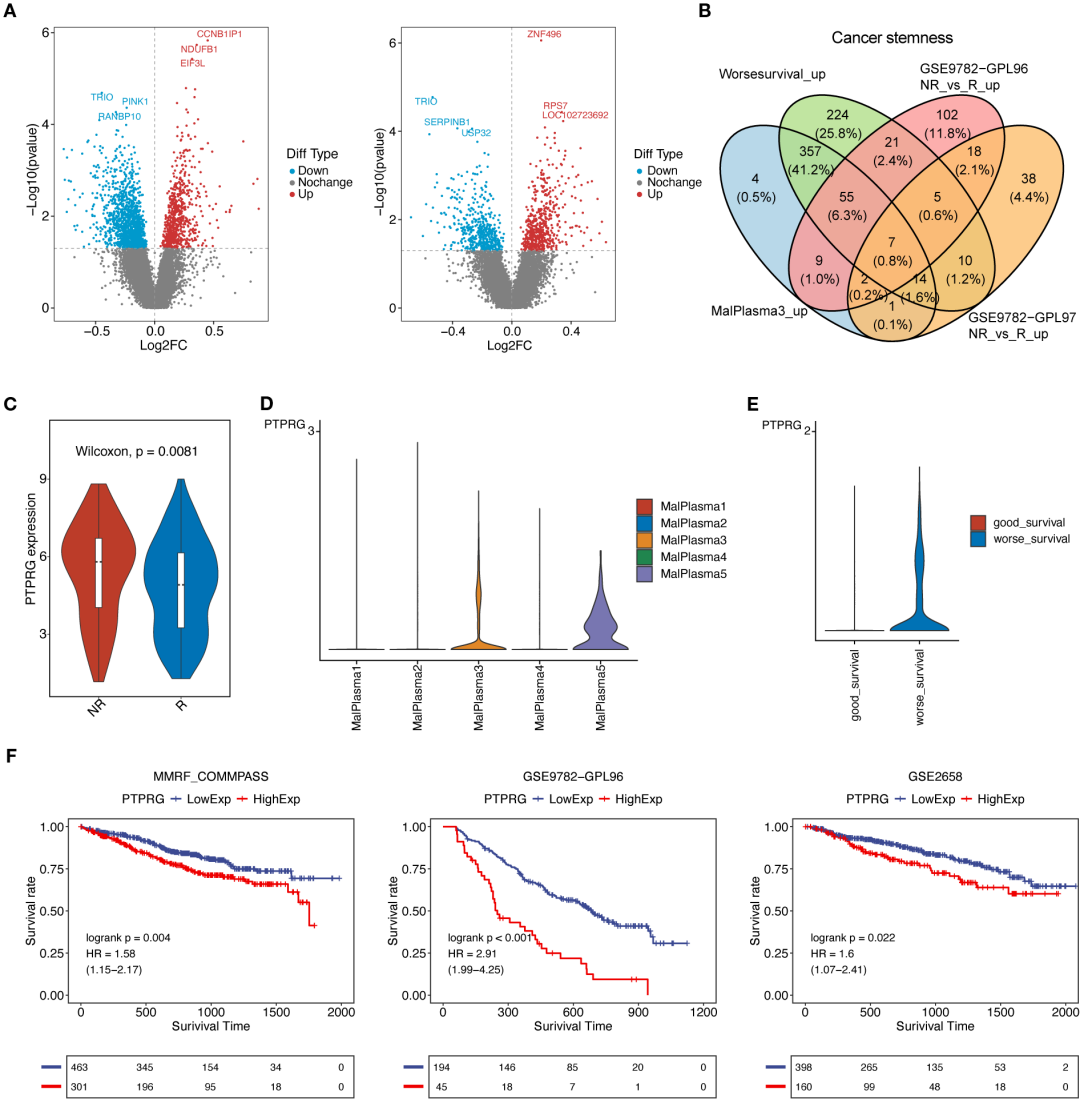
PTPRG affects prognosis and drug resistance. **(A)** Volcano plots of differentially expressed genes (tumor vs normal) in the GSE9782 dataset: (left) GPL96 platform data, (right) GPL97 platform data. **(B)** Venn diagram for obtaining key genes. **(C)** Violin plot of PTPRG expression levels in the GSE9782 dataset (GPL96 platform). **(D)** Violin plot of PTPRG expression levels in the 5 malignant plasma cell subclusters. **(E)** Violin plot of PTPRG expression levels in good survival and worse survival malignant plasma cells. **(F)** Prognostic analysis of PTPRG in bulk datasets. A p-value of <0.05 from the Log-rank test was considered statistically significant.

We further leveraged bulk transcriptomics data from GSE9782 (parallel detection using GPL96 and GPL97 platforms) to assess the relationship between tumor stemness-related genes and prognosis. As shown in Fig8A, a total of 2298 differentially expressed genes (DEGs) were identified in the GPL96 platform data in tumor samples, of which 785 genes were upregulated and 1513 genes were downregulated. In the GPL97 platform data, 1119 DEGs were identified in tumor samples, with 613 genes upregulated and 506 genes downregulated (**Figure 8A**) (P<0.05). Furthermore, we mapped the upregulated DEGs from the MalPlasma3 subcluster (compared to other malignant plasma cell subclusters, a total of 1485 upregulated DEGs) and worse survival malignant plasma cells (compared to good survival malignant plasma cells, a total of 2679 up DEGs) with Cancer stemness pathway genes. We found that Cancer stemness pathway related DEGs were upregulated, including 449 genes in the MalPlasma3 subcluster and 693 genes in worse survival malignant plasma cells. Finally, these genes were overlapped with the upregulated DEGs from the two GSE9782 datasets, resulting in 7 tumor stemness-related genes, including NONO, CBX3, SLC25A3, PTPRG, NPM1, HINT1, HNRNPA1 (**Figure 8B**). Among the 7 genes, PTPRG was specifically highly expressed only in MalPlasma3 and MalPlasma5 subclusters (**Figure 8D**). Considering factors such as whether candidate genes were highly expressed in the responder group (i.e., R group) of the bulk dataset (GSE9782) and in worse survival malignant plasma cells, as well as poor prognosis, PTPRG was ultimately selected. Notably, we found that PTPRG was highly expressed in the NR group of the GSE9782 dataset (GPL96 platform) (**Figure 8C**) and in worse survival malignant plasma cells (**Figure 8E**). Further prognostic validation of PTPRG using bulk datasets GSE9782(best cutoff:6.8011457193872), GSE2658 (best cutoff:9.417325114), and MMRF-CoMMpass (best cutoff: 2.119787759) revealed that samples with high PTPRG expression had significantly poorer prognosis (**Figure 8F**, **Supplementary Figure S3**).

### 3.8 PTPRG affects tumor cell function

**Figure 9:**
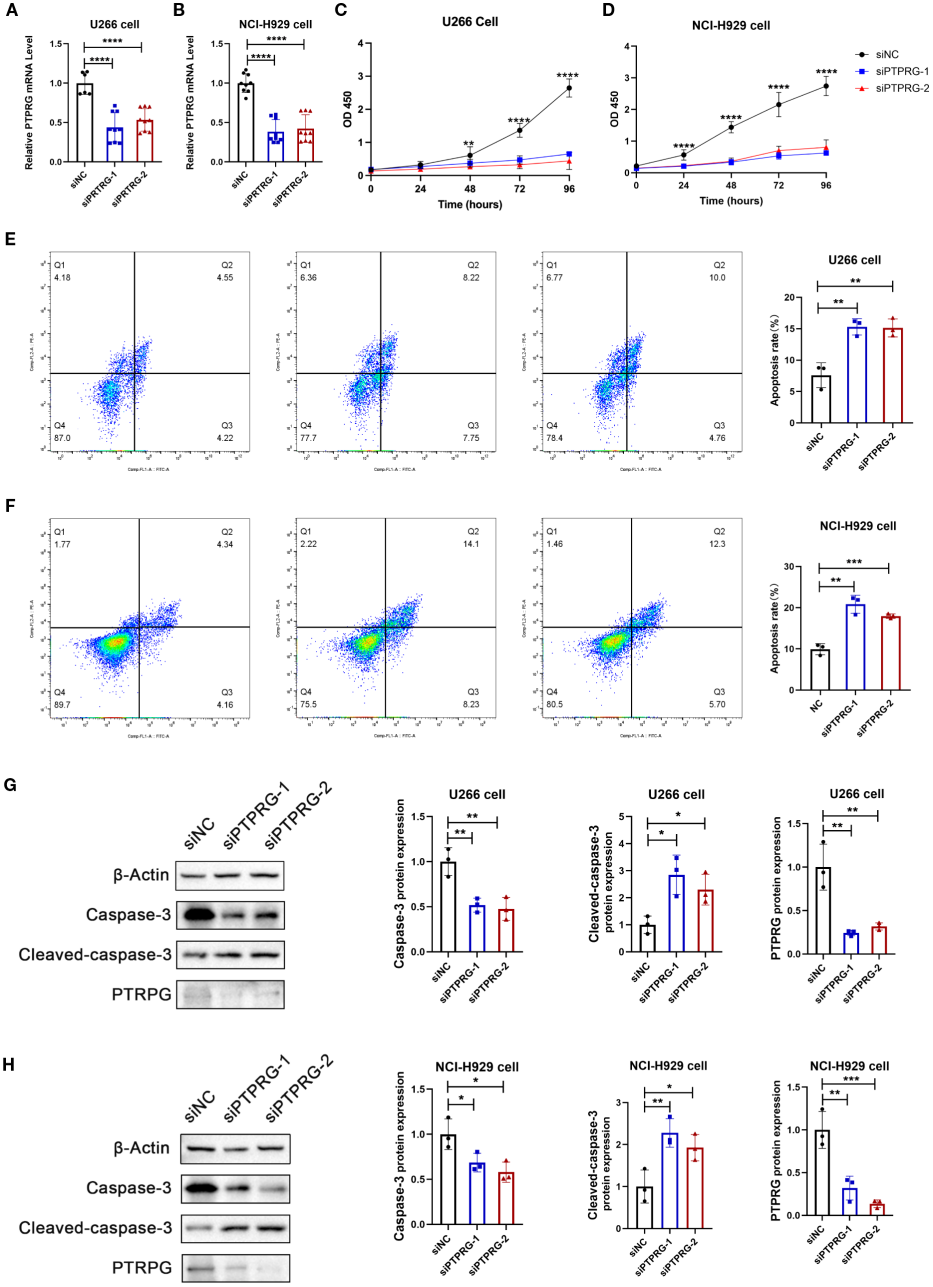
Functional validation of PTPRG. **(A, B)** mRNA expression analysis of PTPRG. Two siRNAs targeting PTPRG were transfected into MM cell lines U266 **(A)** and NCI-H929 **(B)**, followed by RT-qPCR to analyze PTPRG mRNA expression. All data are expressed as mean ± SD. **P<0.01, ***P<0.001, vs si-NC group. **(C-D)** Cell viability analysis. Two siRNAs targeting PTPRG were transfected into MM cell lines U266 **(C)** and NCI-H929 **(D)**, followed by CCK8 assay to analyze cell viability in U266 (left) and NCI-H929 (right). All data are expressed as mean ± SD. ***P<0.001, vs si-NC group. **(E-F)** Cell apoptosis analysis. Annexin V-FITC/PI staining fluorescence-activated cell sorting analysis of MM cell lines U266 **(E)** and NCI-H929 **(F)**; bar chart shows apoptosis rate. All data are expressed as mean ± SD. **P<0.01, ***P<0.001, vs si-NC group. **(G-H)** Apoptotic protein expression analysis. Two siRNAs targeting PTPRG were transfected into MM cell lines U266 and NCI-H929, followed by immunoblotting to analyze protein expression of Caspase-3, Cleaved-caspase-3, and PTPRG in U266 **(G)** and NCI-H929 **(H)**. All data are expressed as mean ± SD. *P<0.05, **P<0.01, ***P<0.001, vs si-NC group.

To better reveal potential functional role of PTPRG in MM, we performed *in vitro* knockdown studies using two distinct siRNAs in U266 and NCI-H929 cell lines. Effective suppression of PTPRG expression was confirmed by qRT-PCR (**Figure 9A, B**), and subsequent CCK-8 assays revealed a significant reduction in cell viability upon PTPRG knockdown (**Figure 9C, D**). Consistently, both flow cytometric analysis and Western blotting demonstrated an increased apoptotic fraction in PTPRG-depleted cells (**Figure 9E–H**), indicating that PTPRG loss inhibits proliferation and promotes apoptosis in MM.

## 4 Discussions

MM remains a formidable hematologic malignancy, characterized by significant inter- and intra-tumoral heterogeneity that profoundly influences therapeutic response and patient outcomes. Our scRNA-seq analysis provides a comprehensive view of the cellular and molecular dynamics in MM, shedding light on the roles of malignant plasma cell heterogeneity and the TME in shaping treatment response and prognosis. By combining single-cell insights with bulk transcriptomics and functional assays, we identified critical cellular subclusters (e.g., a stem‐like plasma‐cell subcluster) and highlighted *PTPRG* as a novel regulator of MM progression.

The diversity of malignant plasma cells in MM is a well-recognized driver of disease complexity and therapeutic challenges. A central finding of our investigation is the delineation of distinct malignant plasma cell subclusters, with the MalPlasma3 subcluster notably enriched in patients exhibiting SOR and strongly associated with a poor prognosis. This aligns with previous work showing that clonal heterogeneity in MM contributes to aggressive disease behavior and resistance to therapies like proteasome inhibitors (24, 25). The prevalence of MalPlasma3 in the SOR group suggests this subpopulation may harbor intrinsic features conferring resistance to the administered therapy or a heightened capacity for adaptive resistance. The enrichment of pathways such as E2F and MYC targets in this subcluster echoes reports that these molecular drivers underpin MM cell survival and rapid growth (26, 27). This is further corroborated by our pseudotime analysis, which depicted a developmental trajectory where malignant plasma cells, particularly those enriched in the SOR group and within MalPlasma3, progress towards a more aggressive, “worse survival” phenotype. Such clonal evolution, where more aggressive or resistant subclones expand, is a known characteristic of MM progression and relapse (28, 29). The enrichment of G2M checkpoint pathways further indicates active cell cycling, a hallmark of aggressive tumor behavior. The identification of E2F8, E2F7, FOXM1, E2F1, and TIMELESS as top active transcription factors in MalPlasma3 provides specific regulatory nodes potentially driving these aggressive characteristics. These insights refine our understanding of how specific malignant subclusters influence MM outcomes, pointing to the need for targeted strategies to address this heterogeneity.

The immune microenvironment is increasingly recognized as a determinant of MM treatment success. Our analysis revealed distinct T-cell subcluster profiles between OR and SOR, with higher proportions of naïve and helper T cells in the OR group. This suggests that a more active immune response may enhance treatment efficacy, consistent with studies linking effective anti-tumor immunity to improved MM (30, 31). Naïve T cells, capable of differentiating into potent effector cells, and helper T cells, which coordinate immune activity, likely bolster anti-tumor defenses (32). The relative scarcity of memory T cells in the OR group could reflect their transition into effector states during therapy, a process known to amplify immune attack (33). These shifts in T-cell composition highlight the adaptability of the immune landscape in MM and its potential as a therapeutic lever, particularly for patients with suboptimal responses.

Interactions within the TME further shape MM progression and response to treatment. Our findings point to enhanced communication between malignant plasma cells and HSPCs in the OR group, mediated by pathways like IGF1-IGF1R. The enrichment of this interaction in the OR group, specifically involving the “poor-prognosis” MalPlasma3 cells, is complex. However, this interaction may foster a supportive bone marrow niche, aiding immune reconstitution and treatment sensitivity (34). It’s conceivable that this interaction, while typically pro-myeloma, might render MalPlasma3 cells more susceptible to certain therapeutic effects in a specific niche context or influence their metabolic state. This contrasts with the general understanding of IGF-1 signaling promoting MM cell fitness (35) and warrants deeper investigation. Additionally, cytokine-driven interactions, such as those involving interferon-gamma and CCL28, in the OR group suggest a more robust immune regulatory environment, corroborating the role of cytokines in MM immune responses (36). These communication patterns emphasize how the TME modulates disease behavior and therapeutic outcomes, offering clues for microenvironment-targeted interventions.

One of our main findings is the identification of PTPRG as a key mediator of the MalPlasma3 phenotype and an independent predictor of poor outcome. PTPRG has been characterized as a receptor‐type phosphatase with tumor‐suppressive functions in solid and hematologic malignancies, where its deletion, methylation, or downregulation unleashes oncogenic kinases such as ABL1 (12, 37). In chronic myeloid leukemia, PTPRG hypermethylation drives resistance to tyrosine‐kinase inhibitors, underscoring its role in constraining aberrant growth signals (38). Our demonstration that high PTPRG expression marks the stem‐like, drug‐resistant MalPlasma3 subcluster and that its knockdown impairs proliferation while promoting apoptosis unveils a previously unrecognized function for PTPRG in MM and positions it as both a novel prognostic biomarker and a tractable target for therapy. The apparent contrast between PTPRG’s canonical tumor-suppressive role elsewhere and its adverse prognostic association in MM likely reflects substrate and network context-dependence. Mechanistically, PTPRG likely modulates phosphorylation‐dependent pathways critical for stemness and cell‐cycle control. Its potential interaction with proliferative pathways, like PI3K/Akt or MAPK, aligns with the broader role of tyrosine phosphatases in cancer signaling (39). In the bone-marrow microenvironment, while IGF-1 provided by stromal elements is a well-established driver of myeloma growth and survival via IGF1R-PI3K/ERK signaling (34, 35)and direct IGF1R dephosphorylation by PTPRG has not been demonstrated, PTPRG dephosphorylates and dampens collaborating RTKs (e.g., FGFR family members), thereby possibly lowering shared adaptors (FRS2-RAS–MAPK; PI3K–AKT) (40) and tuning the effective gain of IGF1R signaling. PTPRG-low states would thus be predicted to elevate RTK tone and amplify IGF-1 to common nodes. In parallel, PTPRG acts as a JAK phosphatase (e.g., JAK2 Y1007/Y1008) (41), positioning it upstream of IFN-γ-JAK-STAT1 signaling. Because IFN-γ robustly induces PD-L1 in myeloma plasma cells (42, 43), reduced PTPRG would be expected to prolong/augment STAT1 signaling and enhance PD-L1 induction, whereas higher PTPRG should blunt this response. These clues highlight that PTPRG may co-tune both IGF1–IGF1R/PI3K–ERK and IFN-γ–JAK–STAT1/PD-L1 axes, offering a mechanistic bridge that ties the microenvironmental cues we observe in the OR group to the poor-prognosis biology of MalPlasma3 and nominating PTPRG as both a prognostic marker and a therapeutically tractable node. Of note, it has been shown that R5 receptor–type PTP inhibitors with PTPRG activity (NAZ2329: cell-permeable, allosteric; SCB4380: competitive, effective with liposomal delivery) suppress tumor growth and stem-like properties in glioma models (44); and murine monoclonal antibodies against the PTPRG ectodomain have been generated and used on CML samples, demonstrating surface accessibility and a plausible route for myeloma-directed biologics (12, 37). Nonetheless, this druggability has not been comprehensively explored in MM and therapeutic value of targeting in *PTPRG* will require preclinical studies (target engagement, selectivity, delivery) followed by appropriately designed early-phase clinical trials.

Our study possesses several notable strengths. The application of scRNA-seq provides a high-resolution, unbiased view of cellular heterogeneity and transcriptional states within both the malignant plasma cell compartment and the TME, offering insights often obscured by bulk analytical methods. The identification and functional annotation of the MalPlasma3 subcluster, its association with poor prognosis, and the detailed pathway analyses contribute significantly to understanding MM biology. A key strength is the identification of PTPRG as a gene linked to this aggressive phenotype and poor outcomes, further supported by *in vitro* validation demonstrating its role in MM cell viability and apoptosis. The comprehensive characterization of T/NK cell subclusters and the delineation of specific cell-cell interaction networks provide a more nuanced understanding of the TME’s contribution to treatment response.

Nevertheless, our study is not without limitations. The small sample size of 18 patients, while informative for scRNA-seq, may limit the statistical power for certain sub-analyses and the broader generalizability of some findings to the diverse MM patient population. While our data integration methods appeared robust, scRNA-seq studies can be susceptible to batch effects and technical variability. The findings are based on patients treated with a specific regimen (bortezomib-melphalan-prednisone), and the identified resistance mechanisms or TME alterations might differ with other therapeutic modalities. The intriguing association of high PTPRG expression with poor prognosis alongside its *in vitro* pro-survival role warrants extensive further validation, particularly through *in vivo* studies using patient-derived xenografts from distinct PTPRG-expressing subclones, and in larger, independent patient cohorts to fully elucidate its mechanistic contributions in different MM contexts. The functional consequences of some observed cell proportion changes (e.g., the apparently contradictory HSPC proportions) and the newly identified cell-cell interactions require more direct experimental validation to establish causality and precise mechanisms.

In conclusion, our study provides a detailed single-cell transcriptomic atlas of the MM bone marrow microenvironment in the context of differential treatment responses. We have identified a prognostically significant malignant plasma cell subcluster, MalPlasma3, characterized by activated stemness and proliferation pathways. Our findings highlighted PTPRG as a gene whose high expression is linked to this aggressive phenotype and poor clinical outcomes in MM, yet intriguingly, its knockdown *in vitro* impairs MM cell survival, suggesting a complex, context-dependent pro-survival function in these cells that warrants deeper exploration as a potential therapeutic vulnerability. Furthermore, we delineate distinct TME compositions, particularly within T/NK cell subsets, and specific cell-cell interaction networks involving IGF1-IGF1R, IFNG, and CCL28-CCR10, that are associated with optimal versus suboptimal treatment responses.

## Data Availability

The original contributions presented in the study are included in the article/**Supplementary Material**. Further inquiries can be directed to the corresponding author.

## Supplementary material

Supplementary Figure 1 UMAP visualization technology was used for unsupervised clustering analysis of cells, where each dot represents a single cell. **A** Cells colored by cluster. **B** Dot plot showing the cell counts for different cell types. **C** Shows the cell type proportion in each sample. **D** Shows the proportion of each cell type in the two sample groups. **E** Shows CNV score values. **F** UMAP plot showing CNV score values for plasma cells and T/NK cells.

Supplementary Figure 2 Details of T/NK cell reclustering. **A** UMAP plot showing the distribution of different T/NK cell clusters. **B** UMAP plot showing the distribution of different samples within T/NK cell subclusters. **C** Bar chart showing the cell counts of T/NK cell subclusters. **D** Stacked bar plot showing the proportion of T/NK cell subclusters in each sample. **E** Stacked bar plot showing the proportion of each sample within T/NK cell subclusters. **F** Stacked bar plot showing the proportion of OR and SOR groups within T/NK cell subclusters.

Supplementary Figure 3 The univariate Cox results of the PTPRG gene in three datasets.

Supplementary Table 1 The gene set related to the “cancer stemness” and “Cytotoxicity” function of T cells.

Supplementary Table 2 The target sites of siRNA for PTPRG.

Supplementary Table 3 The primers of β-ACTIN and PTPRG.

Supplementary Table 4 Description of patients and single cell data included in this study.
