# Supplementary figures and images for: Single-cell profiling uncovers PTPRG-driven stemness in malignant plasma cells and signatures of treatment failure in multiple myeloma
Source: Front Immunol. 2025 Sep 18;16:1658028. doi: 10.3389/fimmu.2025.1658028 (PMC12488599; doi:10.3389/fimmu.2025.1658028)

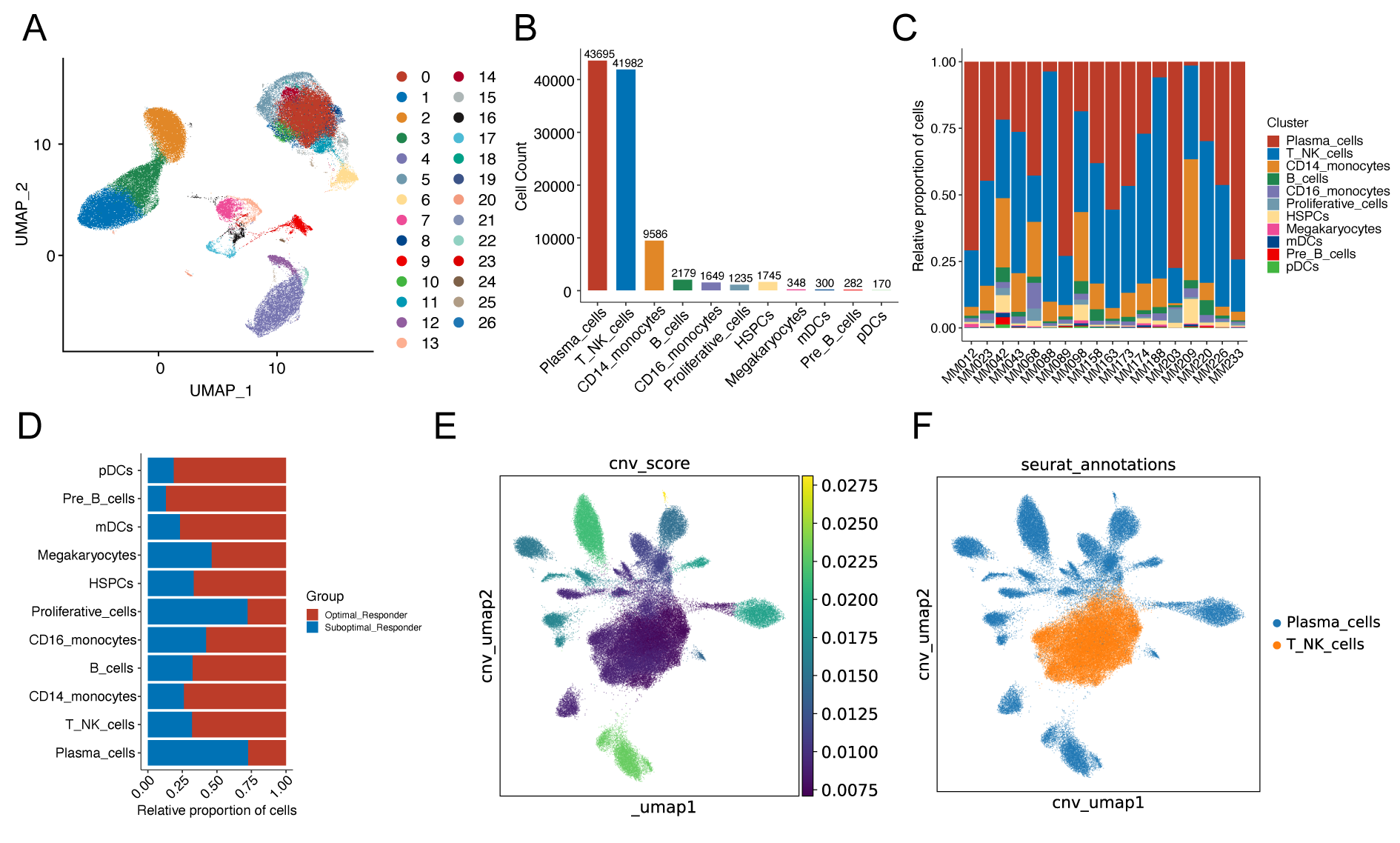

Supplement: Supplementary Figure 1 — UMAP visualization technology was used for unsupervised clustering analysis of cells, where each dot represents a single cell. A Cells colored by cluster. B Dot plot showing the cell counts for different cell types. C Shows the cell type proportion in each sample. D Shows the proportion of each cell type in the two sample groups. E Shows CNV score values. F UMAP plot showing CNV score values for plasma cells and T/NK cells. [file Image1.tif]

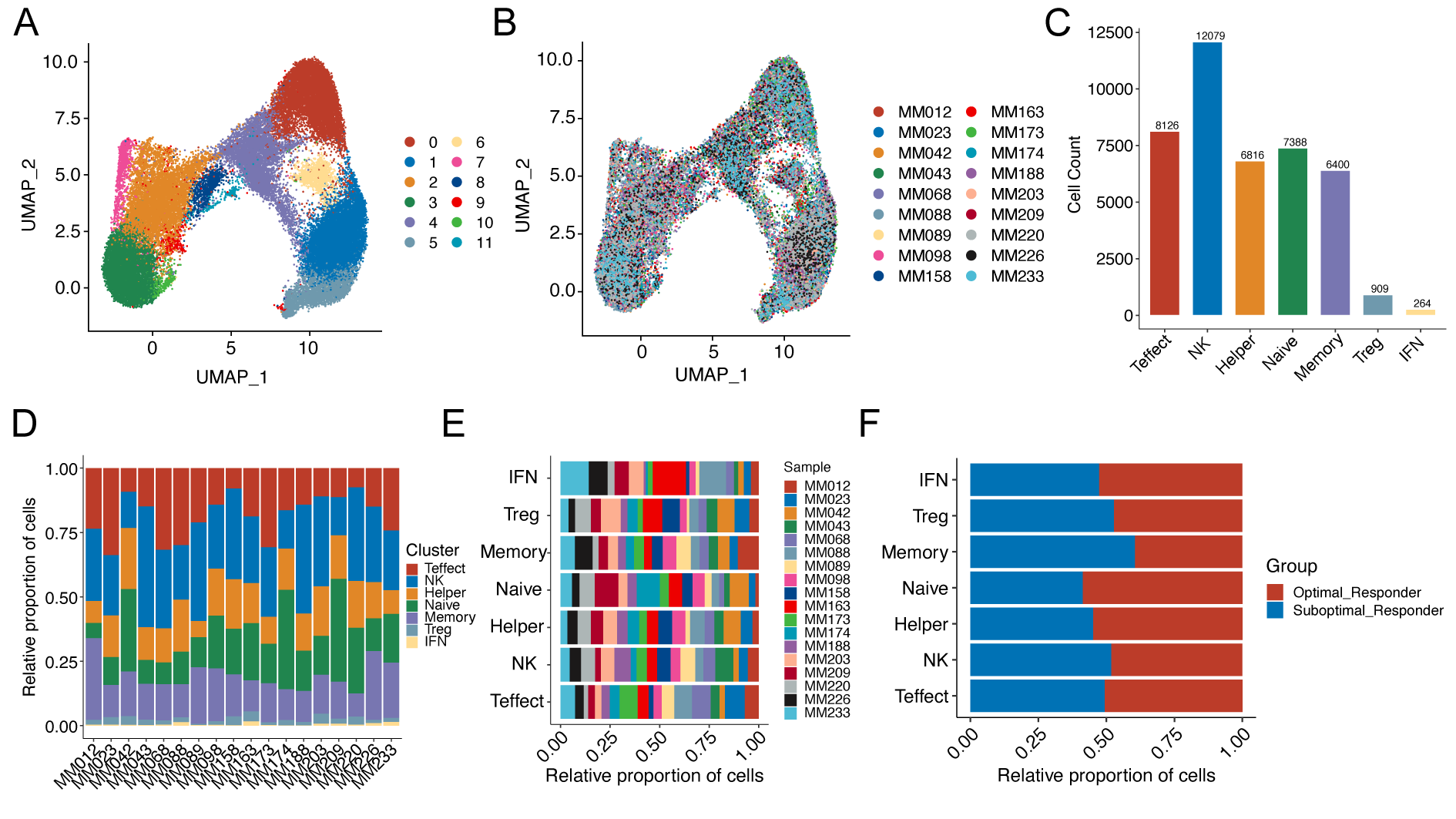

Supplement: Supplementary Figure 2 — Details of T/NK cell reclustering. A UMAP plot showing the distribution of different T/NK cell clusters. B UMAP plot showing the distribution of different samples within T/NK cell subclusters. C Bar chart showing the cell counts of T/NK cell subclusters. D Stacked bar plot showing the proportion of T/NK cell subclusters in each sample. E Stacked bar plot showing the proportion of each sample within T/NK cell subclusters. F Stacked bar plot showing the proportion of OR and SOR groups within T/NK cell subclusters. [file Image2.tif]

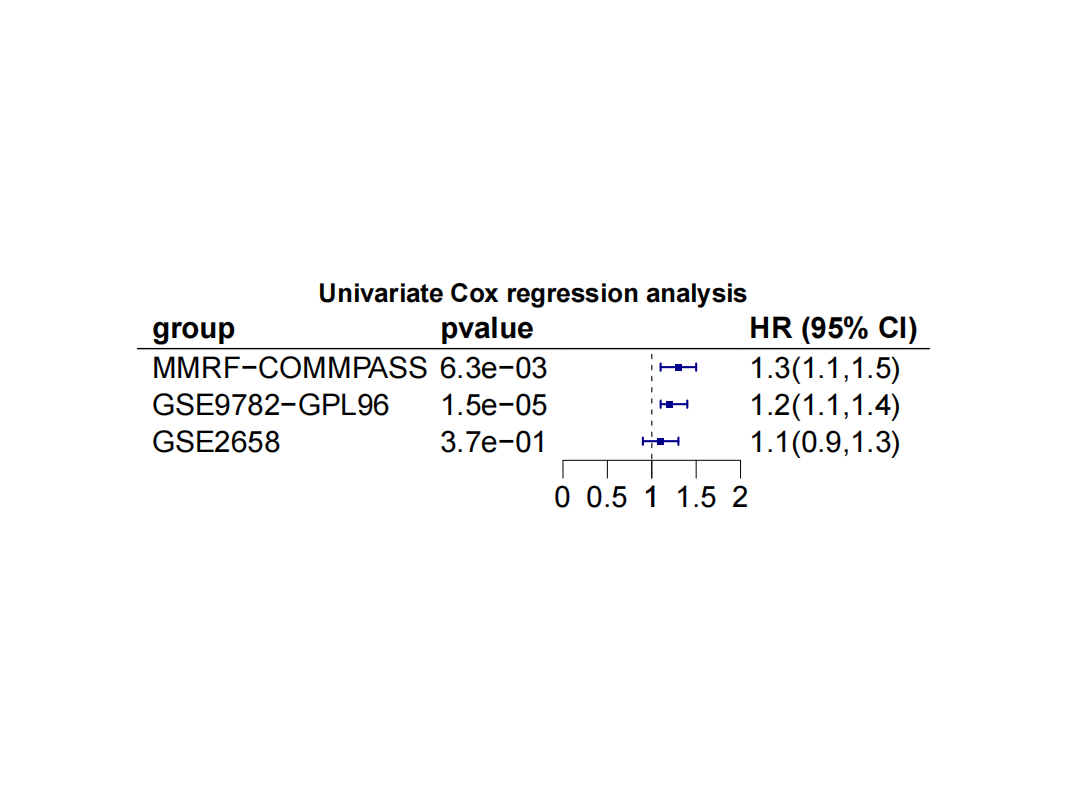

Supplement: Supplementary Figure 3 — The univariate Cox results of the PTPRG gene in three datasets. [file Image3.tif]

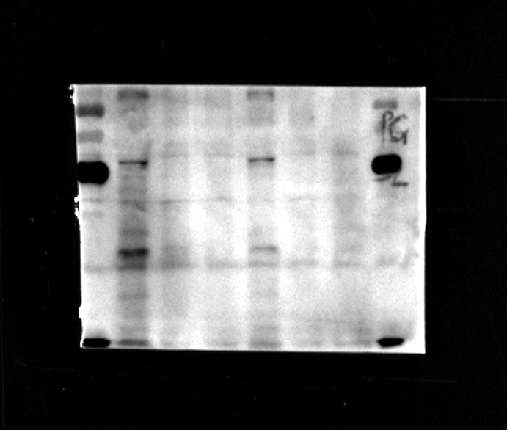

Supplement: Supplementary file 8 [file DataSheet1.zip › the original image files for the blots/PTRPG-3.tif]

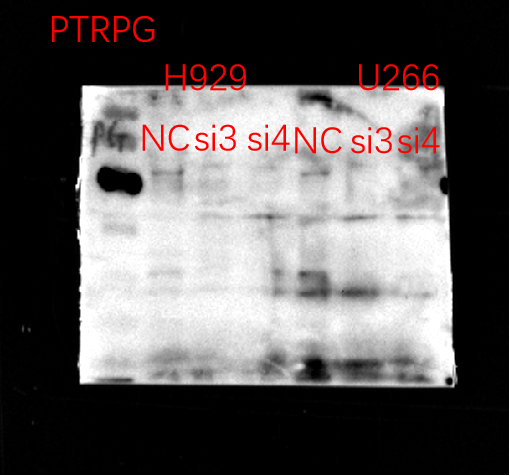

Supplement: Supplementary file 8 [file DataSheet1.zip › the original image files for the blots/PTRPG-2.tif]

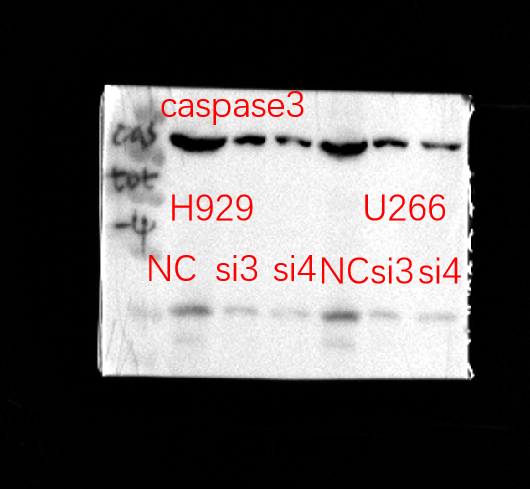

Supplement: Supplementary file 8 [file DataSheet1.zip › the original image files for the blots/caspase-3-2.tif]

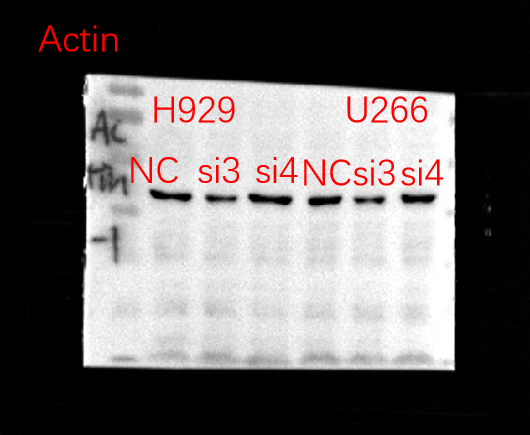

Supplement: Supplementary file 8 [file DataSheet1.zip › the original image files for the blots/Actin-2.tif]

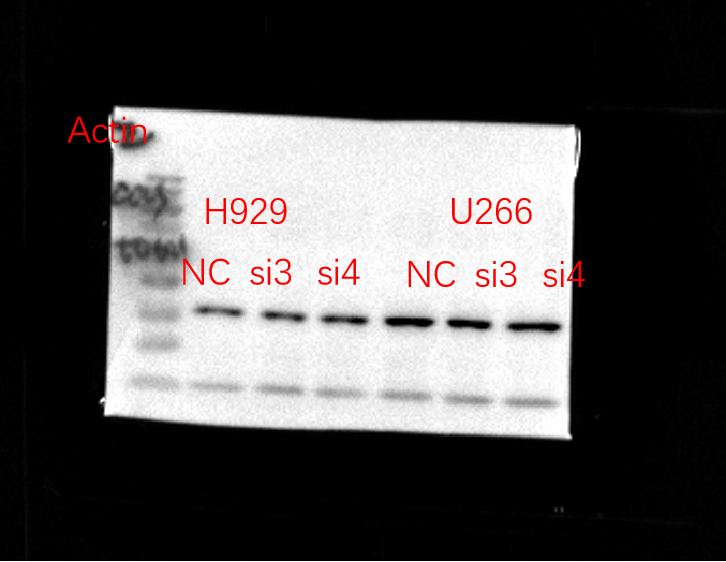

Supplement: Supplementary file 8 [file DataSheet1.zip › the original image files for the blots/Actin-1.tif]

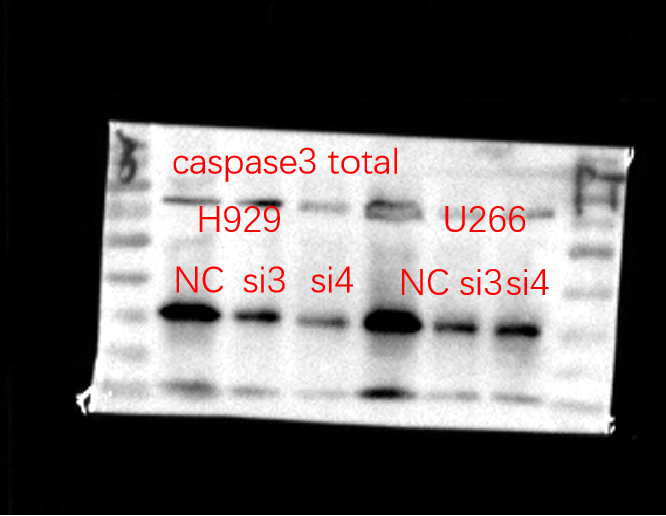

Supplement: Supplementary file 8 [file DataSheet1.zip › the original image files for the blots/caspase-3-1.tif]

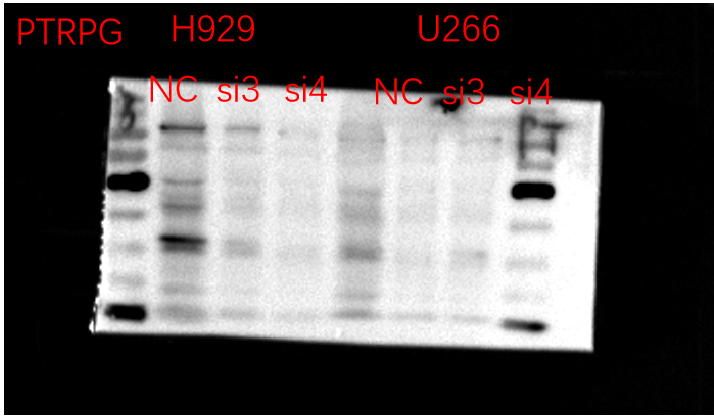

Supplement: Supplementary file 8 [file DataSheet1.zip › the original image files for the blots/PTRPG-1.tif]

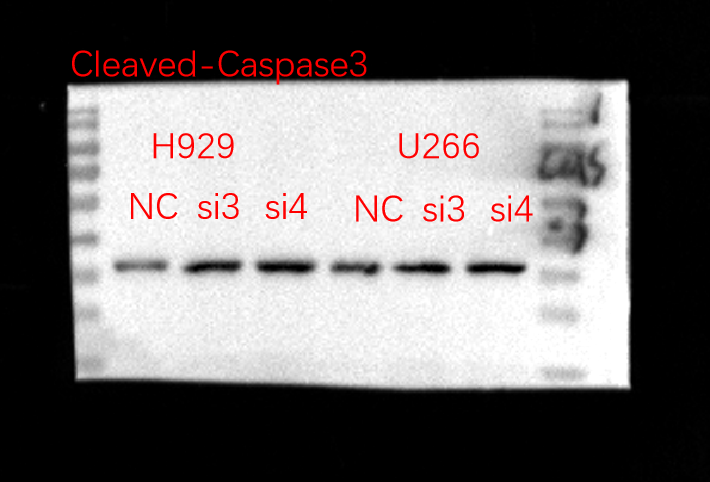

Supplement: Supplementary file 8 [file DataSheet1.zip › the original image files for the blots/Cleaved-caspase-3-1.tif]

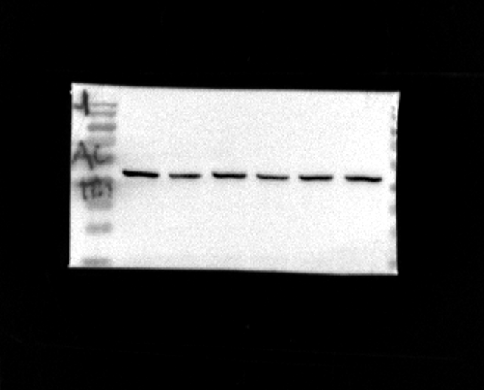

Supplement: Supplementary file 8 [file DataSheet1.zip › the original image files for the blots/Actin-3.tif]

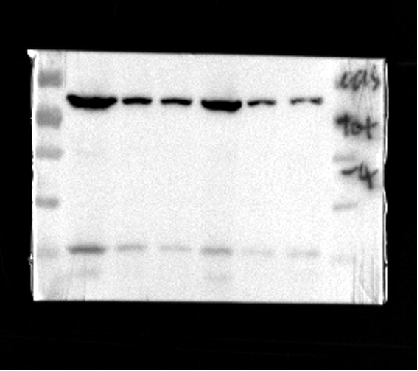

Supplement: Supplementary file 8 [file DataSheet1.zip › the original image files for the blots/caspase-3-3.tif]

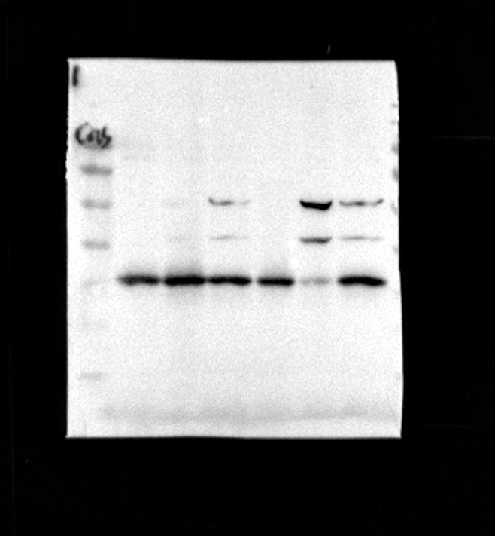

Supplement: Supplementary file 8 [file DataSheet1.zip › the original image files for the blots/Cleaved-caspase-3-3.tif]

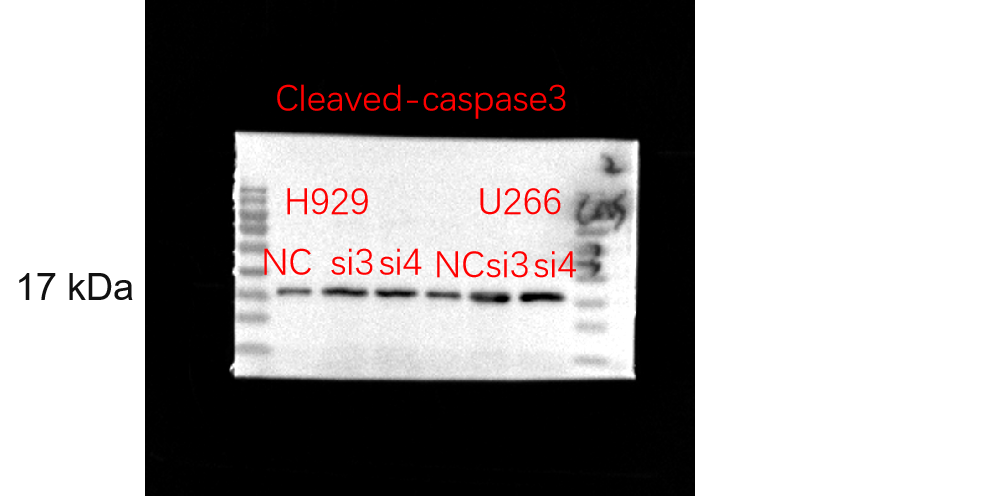

Supplement: Supplementary file 8 [file DataSheet1.zip › the original image files for the blots/Cleaved-caspase-3-2.tif]

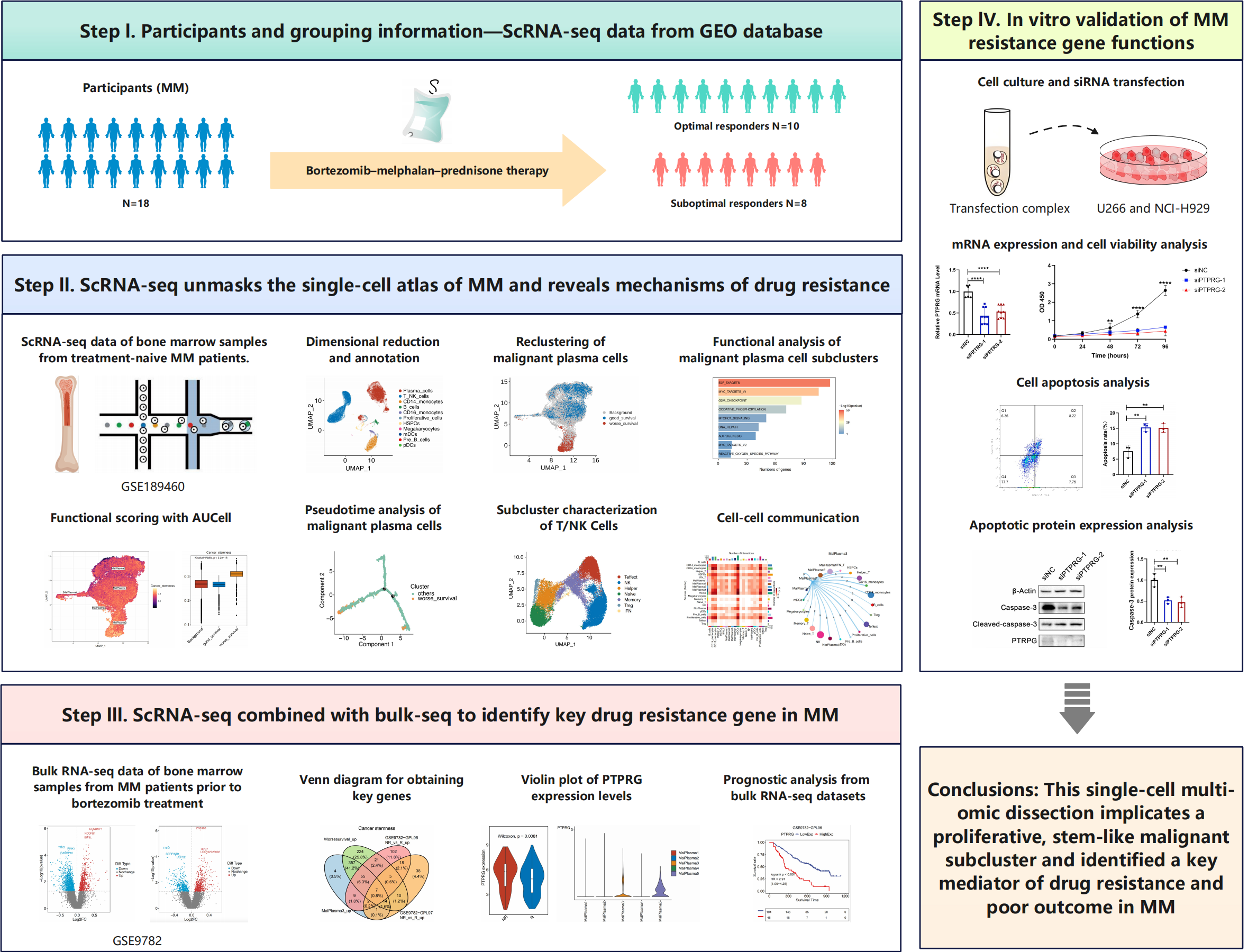

Supplement: Supplementary file 9 [file DataSheet2.zip › Fig/Fig1.tif]

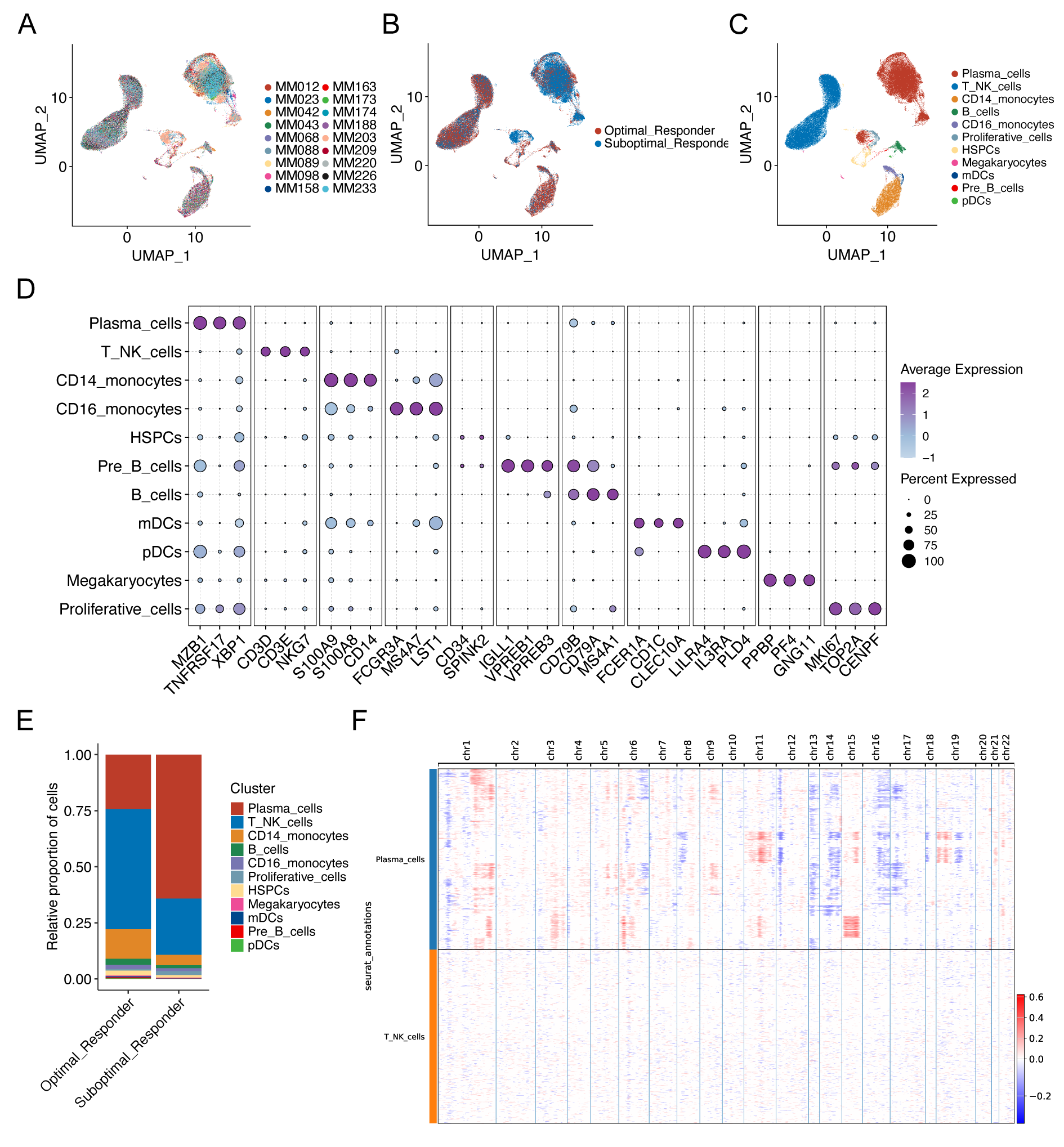

Supplement: Supplementary file 9 [file DataSheet2.zip › Fig/fig2.tif]

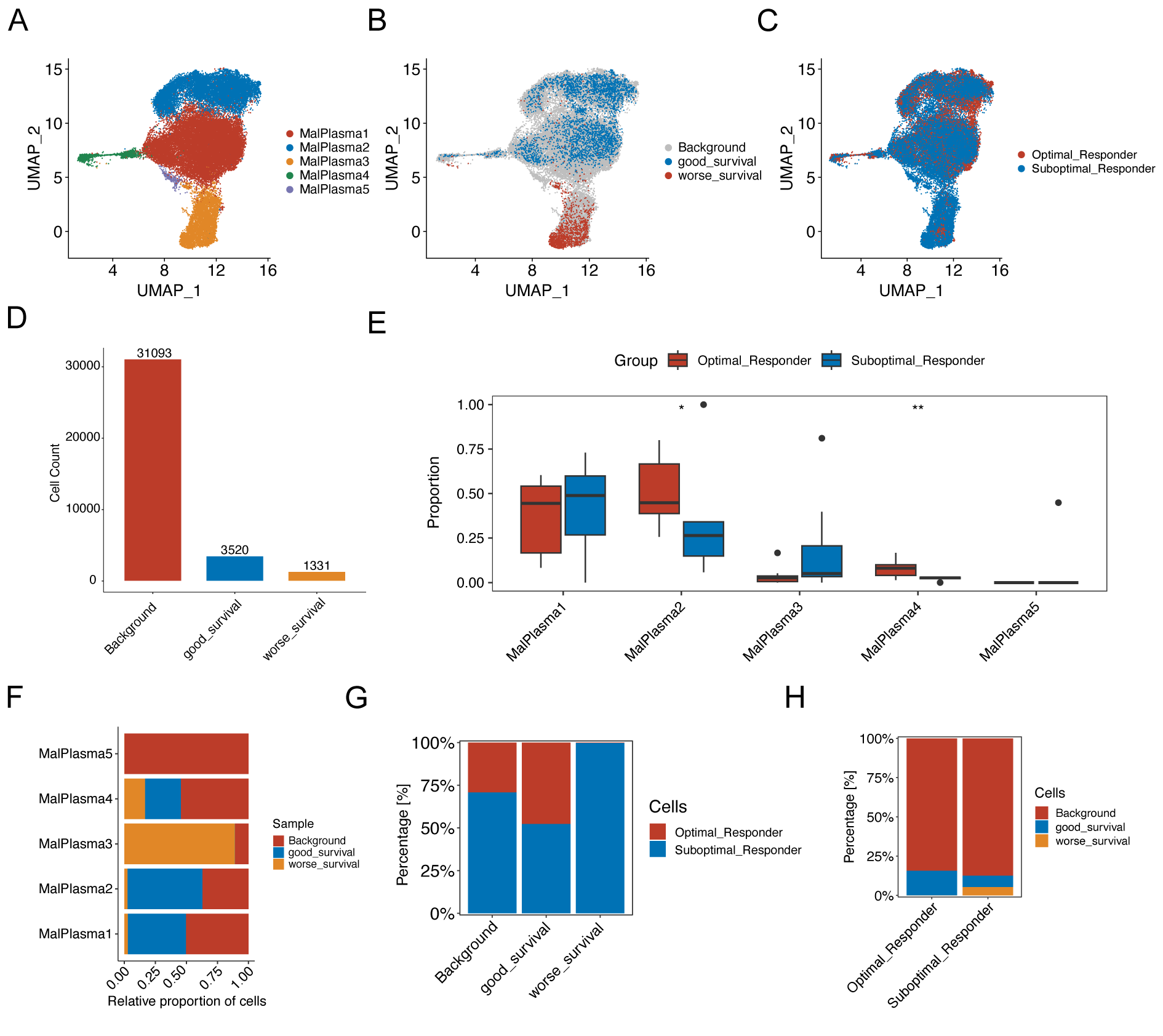

Supplement: Supplementary file 9 [file DataSheet2.zip › Fig/fig3.tif]

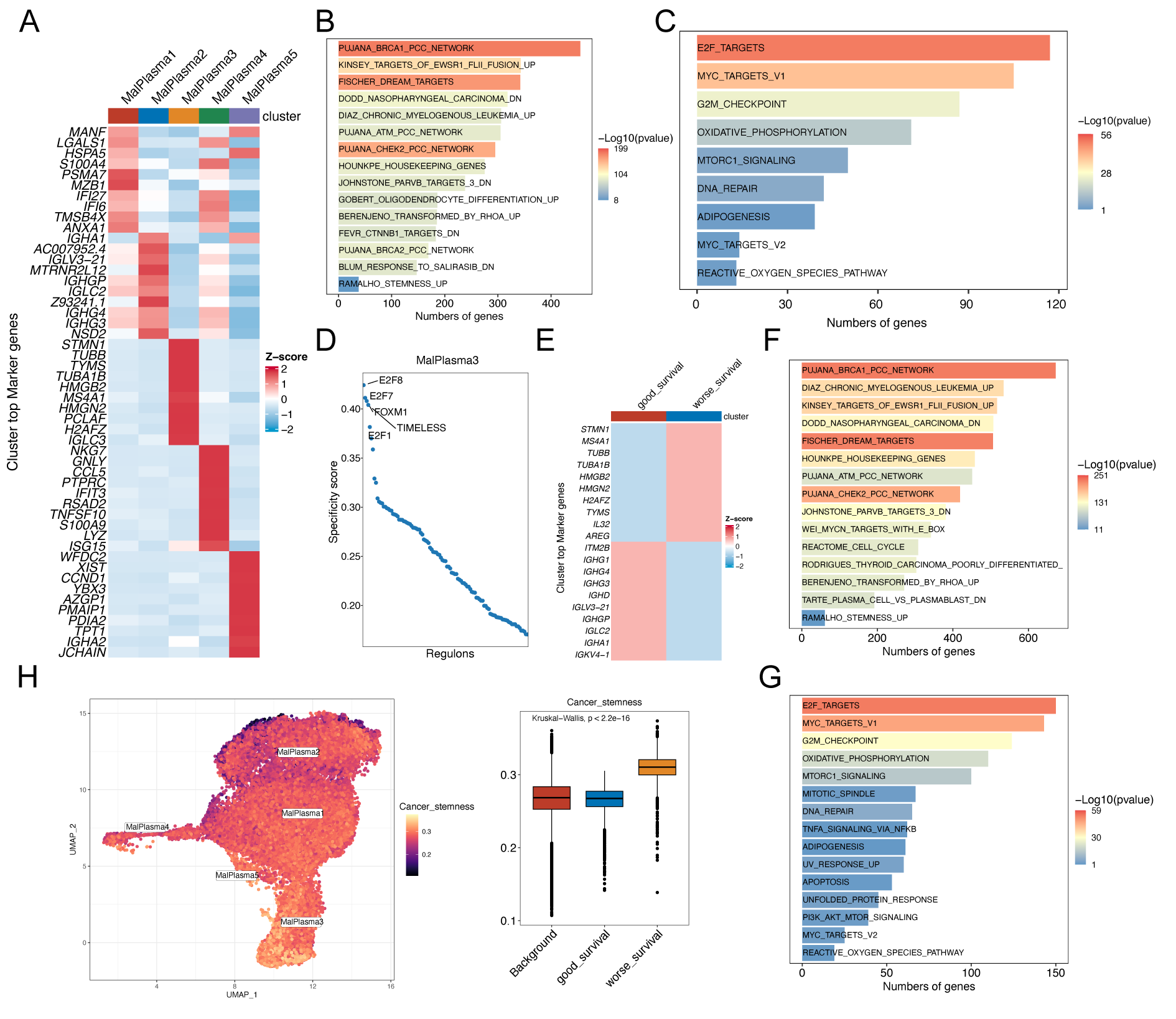

Supplement: Supplementary file 9 [file DataSheet2.zip › Fig/fig4.tif]

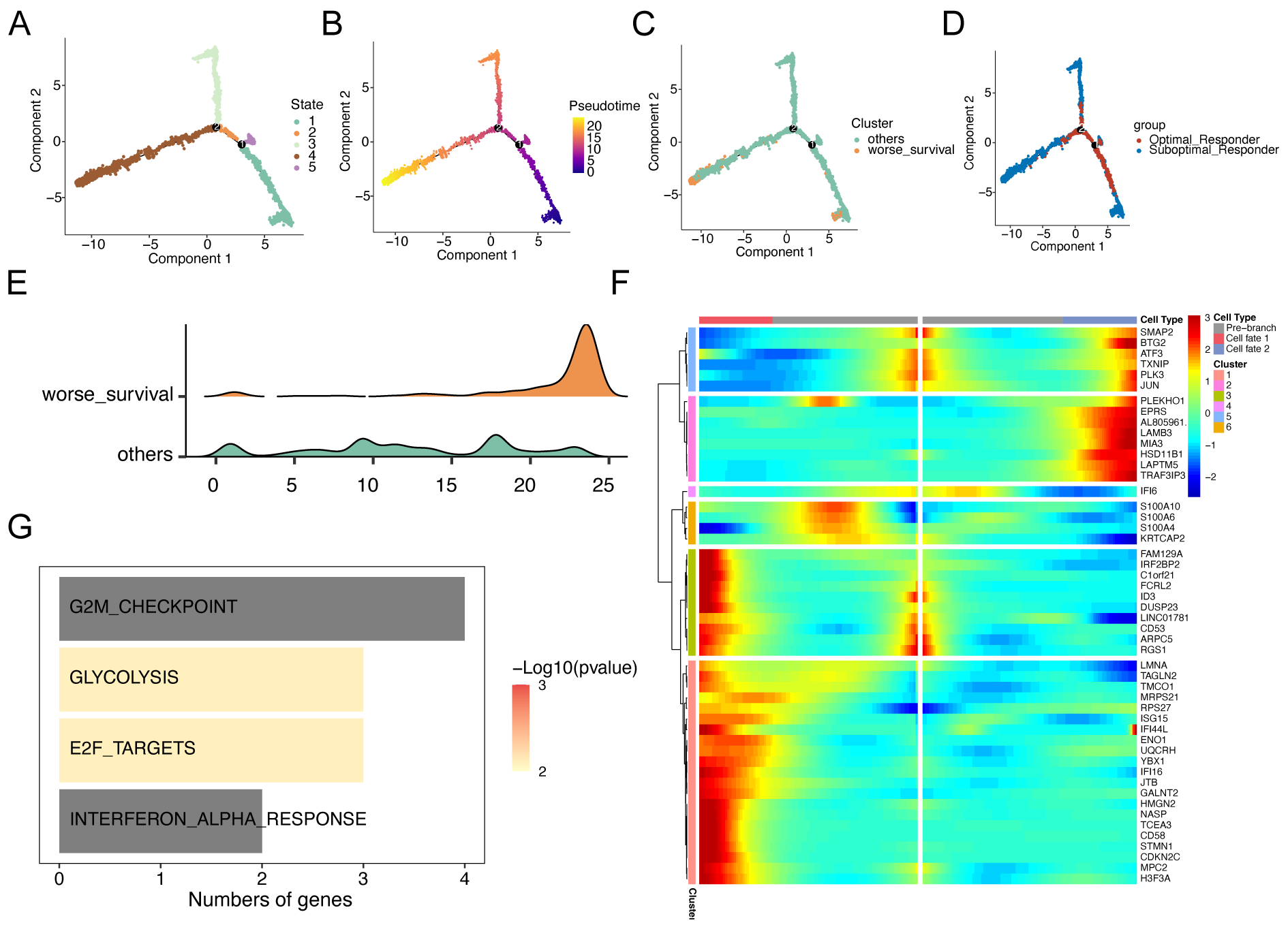

Supplement: Supplementary file 9 [file DataSheet2.zip › Fig/fig5.tif]

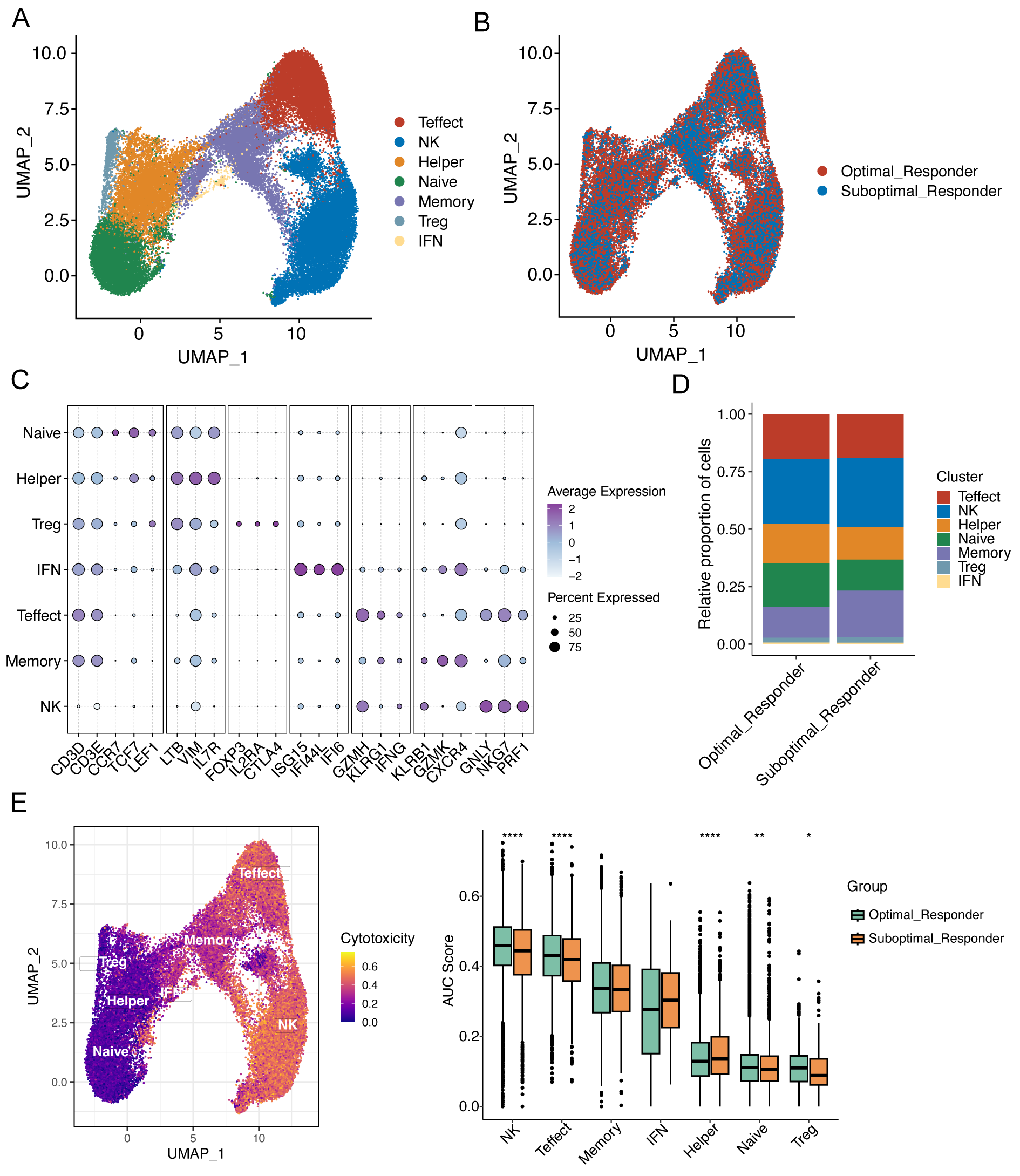

Supplement: Supplementary file 9 [file DataSheet2.zip › Fig/fig6.tif]

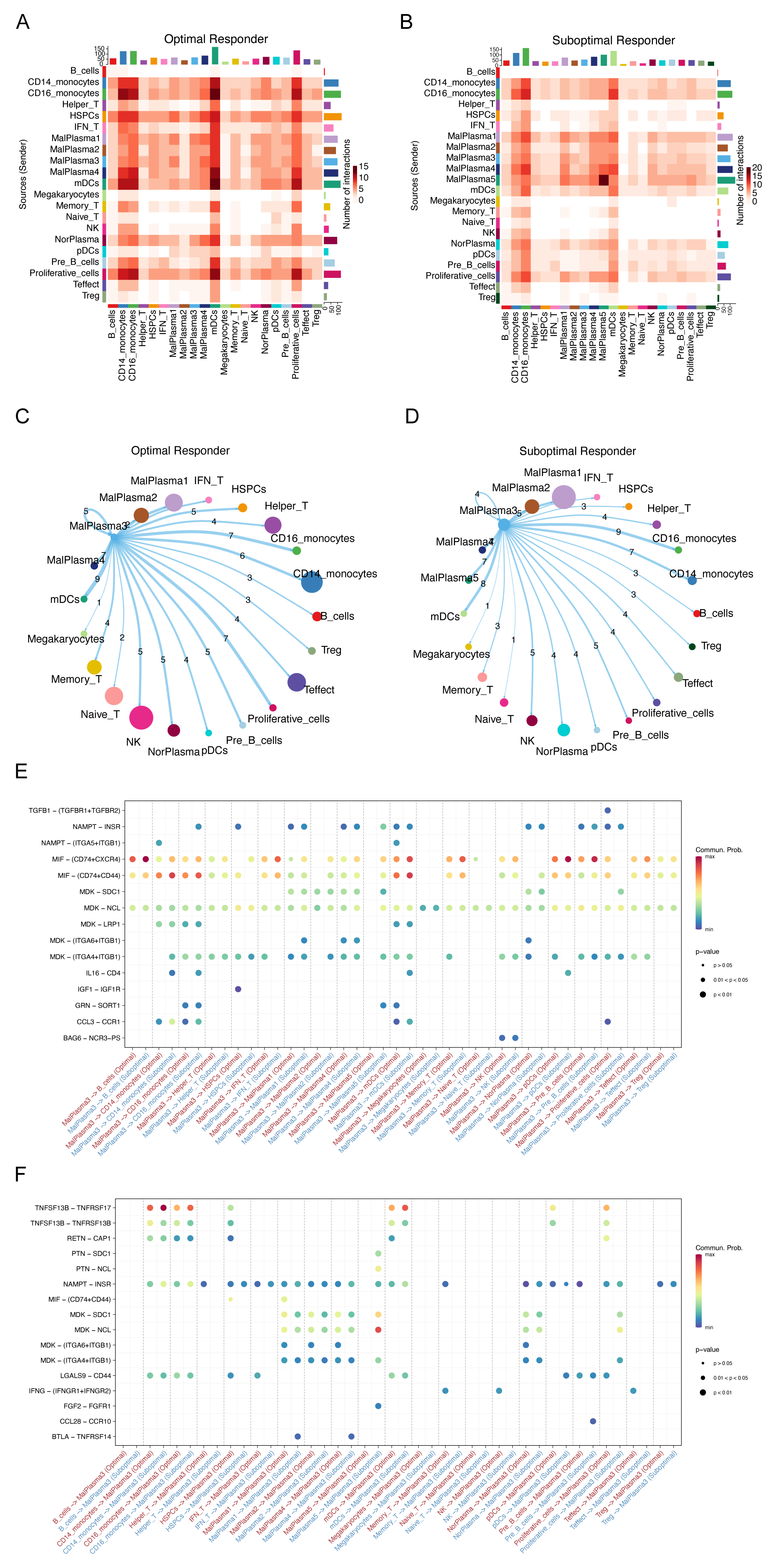

Supplement: Supplementary file 9 [file DataSheet2.zip › Fig/fig7.tif]

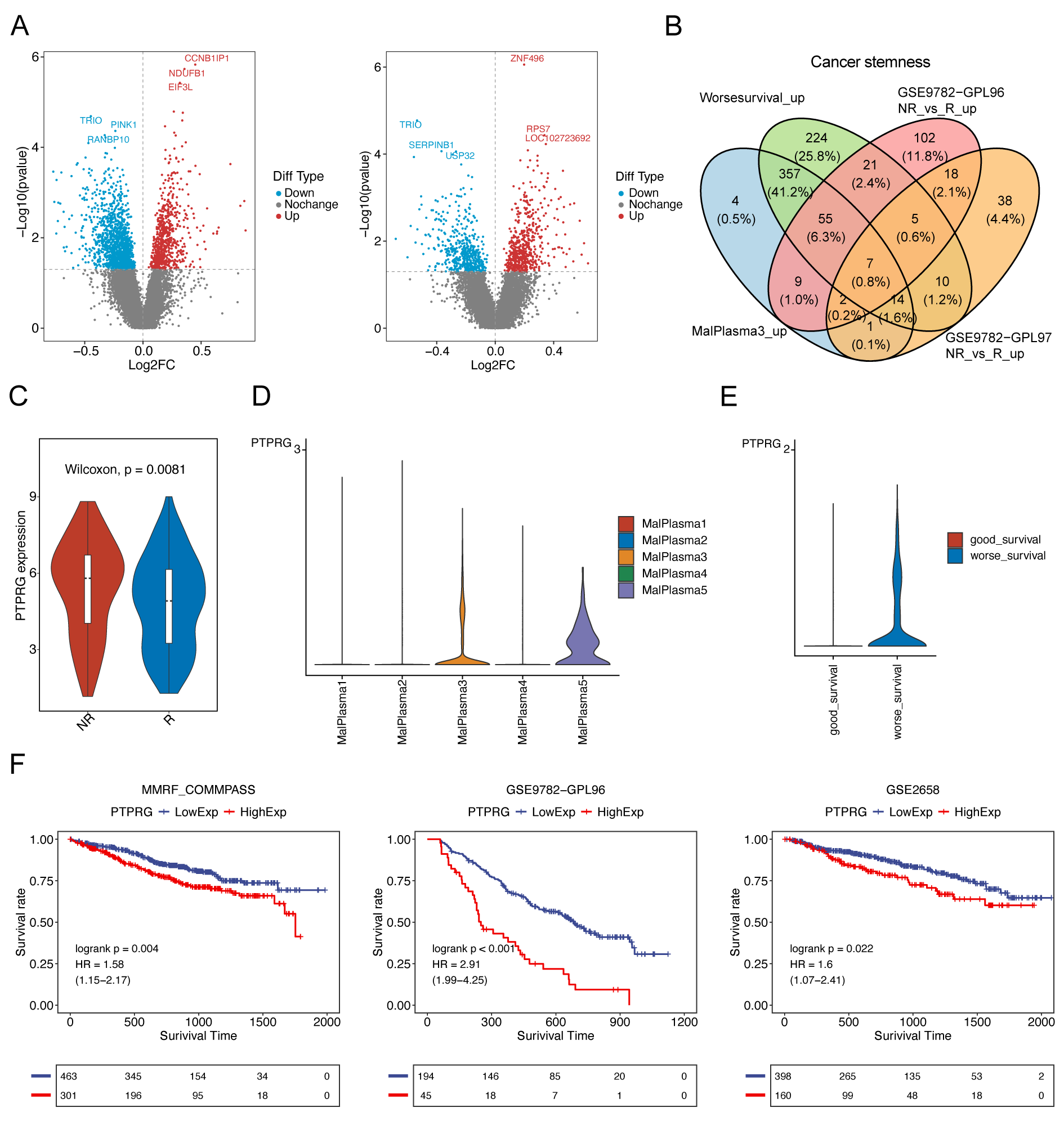

Supplement: Supplementary file 9 [file DataSheet2.zip › Fig/fig8.tif]

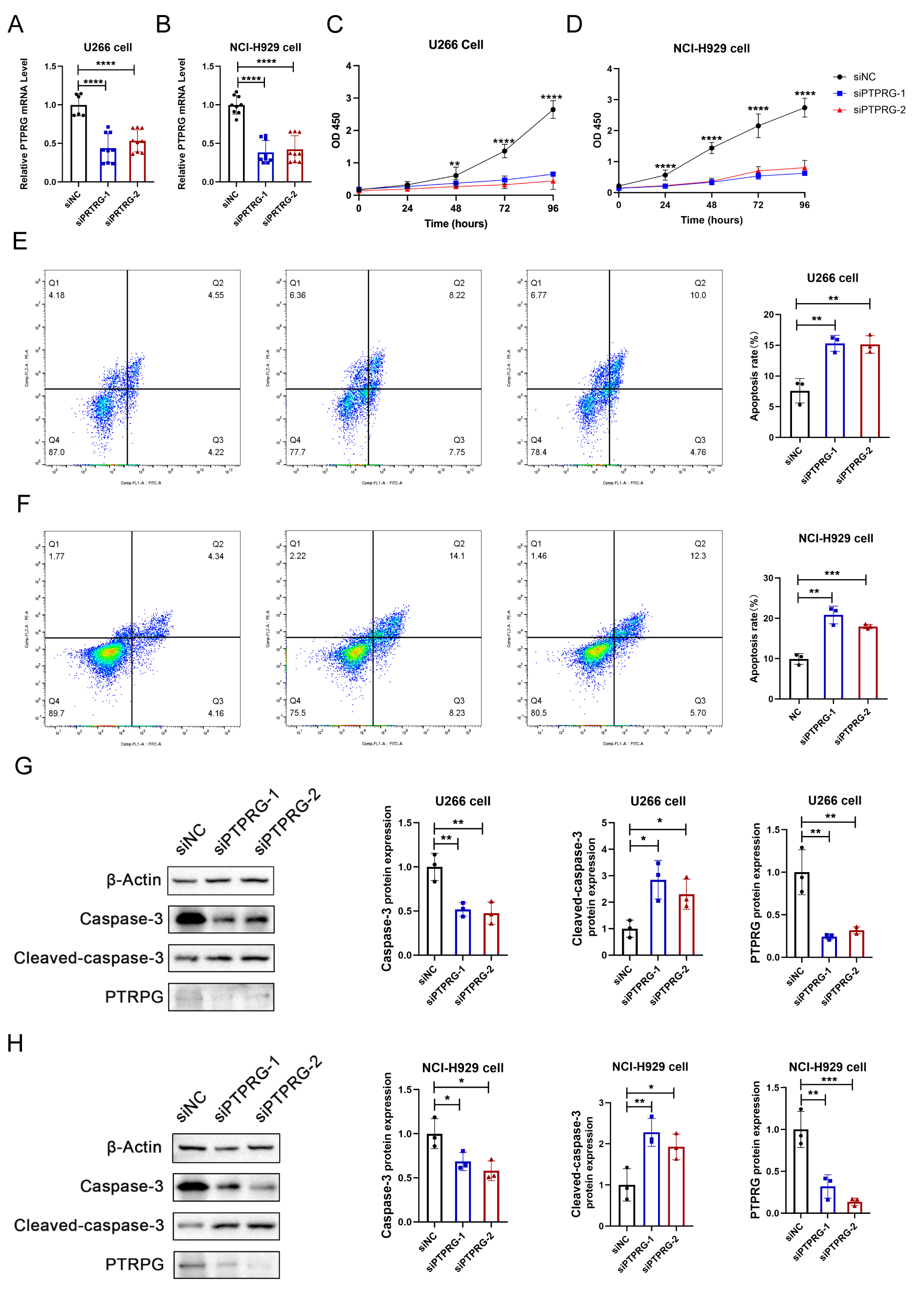

Supplement: Supplementary file 9 [file DataSheet2.zip › Fig/fig9.tif]

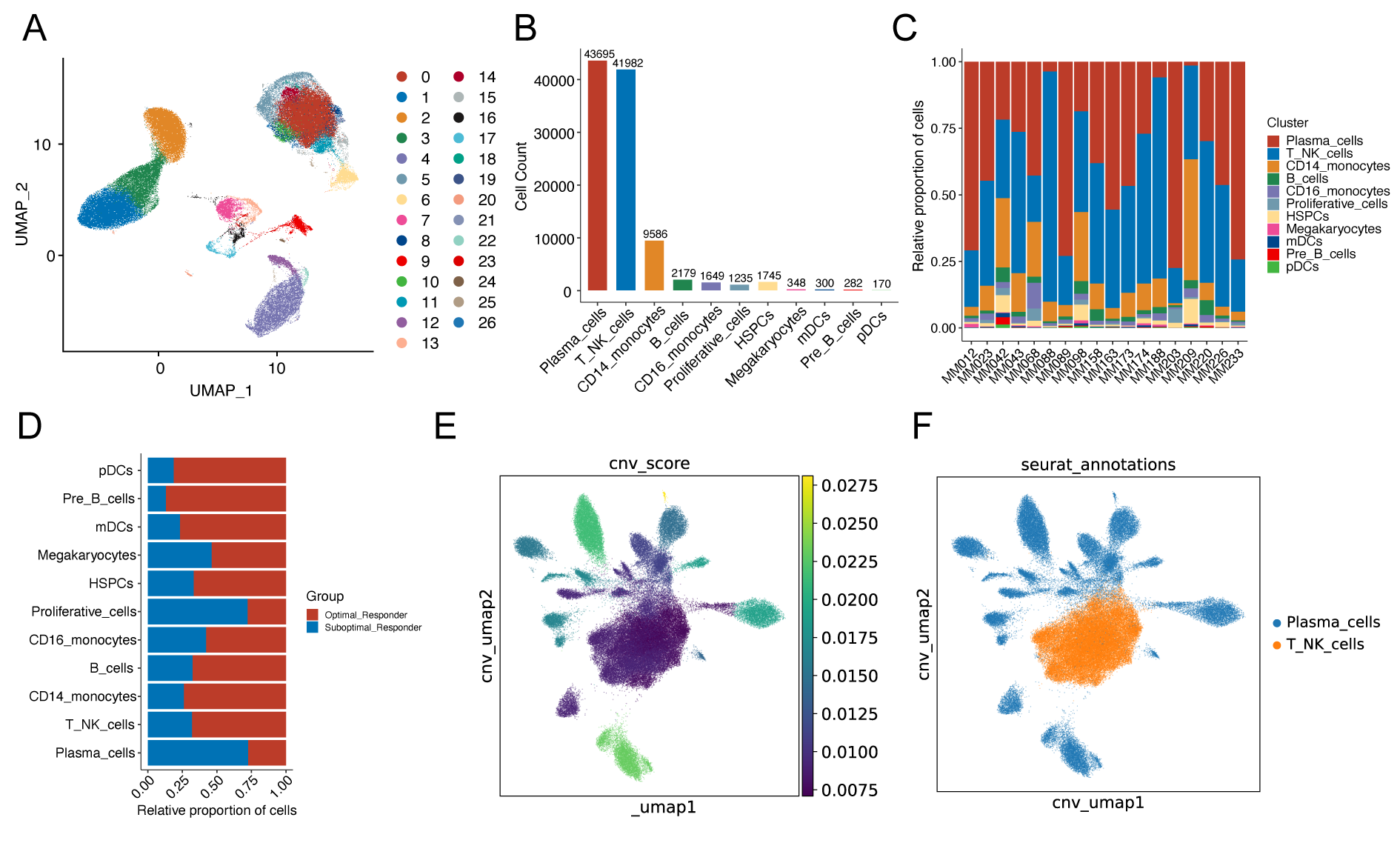

Supplement: Supplementary file 9 [file DataSheet2.zip › Fig/figs1.tif]

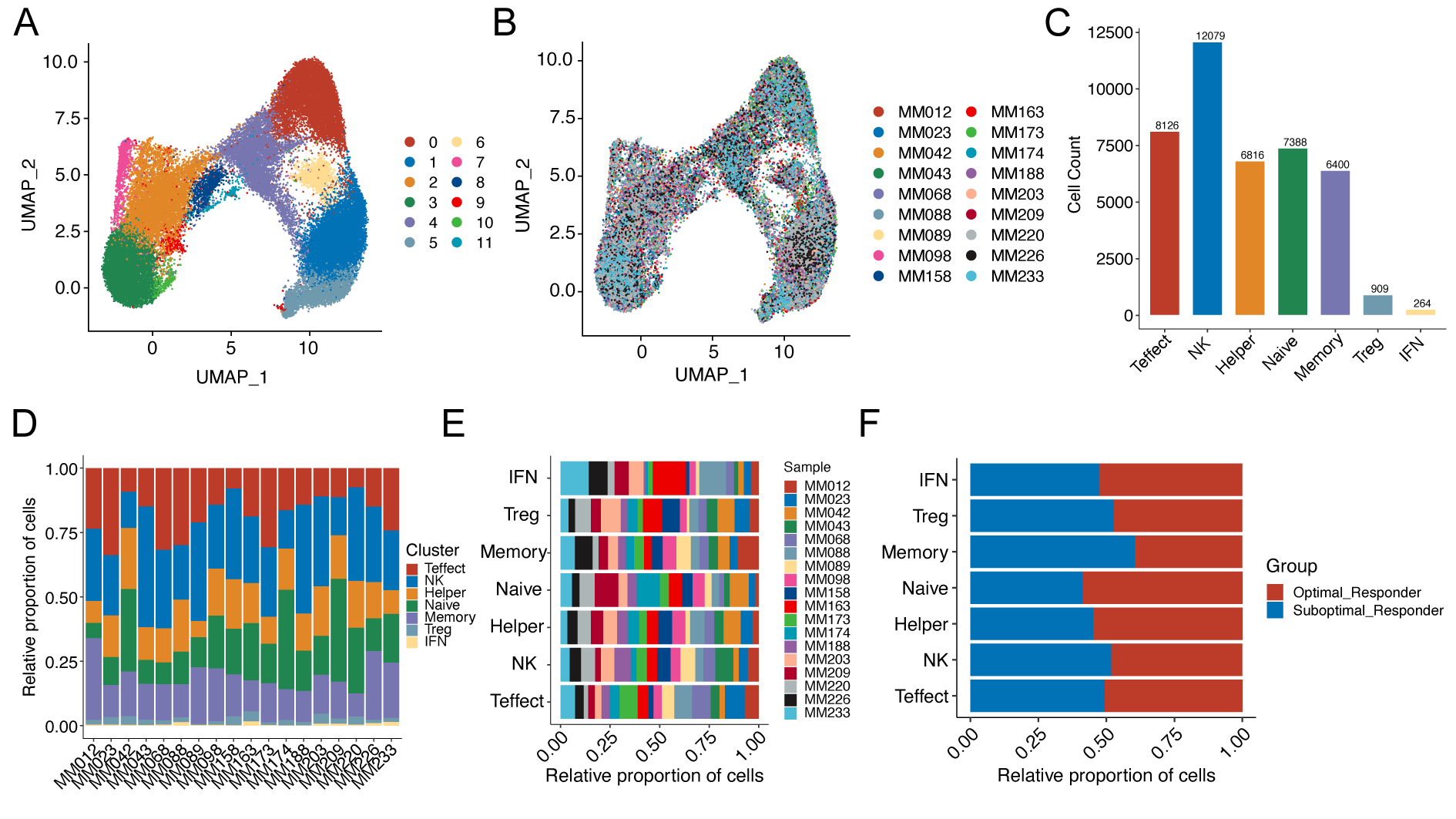

Supplement: Supplementary file 9 [file DataSheet2.zip › Fig/figs2.tif]

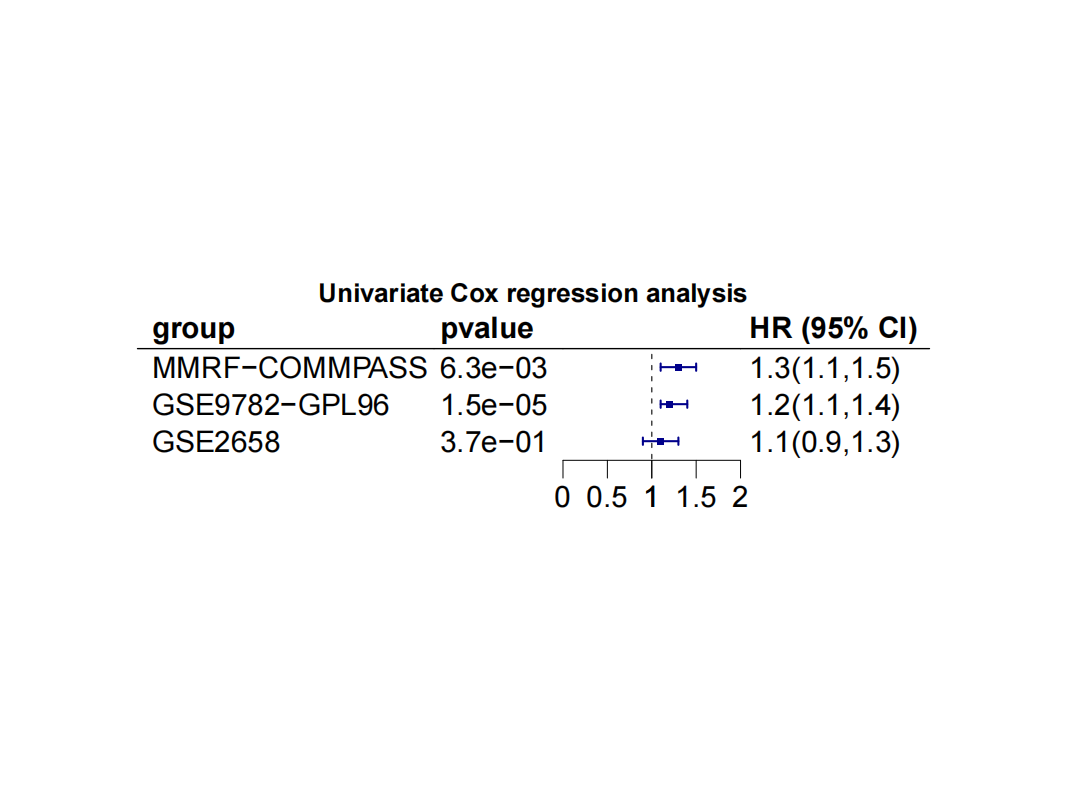

Supplement: Supplementary file 9 [file DataSheet2.zip › Fig/FigS3.tif]
